# Handwriting Moroccan regions recognition using Tifinagh character

**DOI:** 10.1016/j.dib.2015.07.018

**Published:** 2015-07-26

**Authors:** B. El Kessab, C. Daoui, B. Bouikhalene, R. Salouan

**Affiliations:** Laboratory of Information Processing and Decision Support, Morocco

## Abstract

The territorial organization of Morocco during administratives division of 2009 is based on 16 regions. In this work we will create a system of recognition of handwritten words (names of regions) using the Amazigh language is an official language by the Moroccan Royal Institute of Amazigh Culture (IRCAM) (2003a) [1] such as this language is slightly treated by researchers in pattern recognition field that is why we decided to study this language (El Kessab et al., 2013 [3]; El Kessab et al., 2014 [4]) that knowing the state make a decision to computerize the various public sectors by this language.

In this context we propose a data set for handwritten Tifinagh regions composed of 1600 image (100 Image for each region). The dataset can be used in one hand to test the efficiency of the Tifinagh region recognition system in extraction of characteristics significatives and the correct identification of each region in classification phase in the other hand.

Specifications table

**Table:** 

Subject area	Computer science
More specific subject area	Image processing, handwritten Tifinagh region, the Amazigh language
Type of data	Image
How data was acquired	Handwritten, Scanner, Marker
Data format	Jpeg image
Experimental factors	We ask 70 students to write 16 regions with Tifinagh characters, we use an HP G3110 with maximum resolution 4800×9600 dpi to data scan, and we use a marker in writing of characters
Experimental features	1376 Image with a size of 30×30 pixels (100 images/region)
Data source location	Béni Mellal, Morocco
Data accessibility	Within this article

Value of the data

• The region is the current highest administrative division of Morocco. The regions are subdivided into a total of 63s-order administrative divisions, which are prefectures and provinces [2] A Moroccan region is governed by a Wali, nominated by the King. The Wali is also governor of the province (or prefecture) where he resides.
• As part of a 1997 decentralization and regionalization law passed by the legislature 16 new regions of Morocco were created.
• We chose a database word contains 1000 words written in marker and that represents the 16 region of Morocco.
• Optical Character Recognition (OCR) can be applied on both cases printed or handwritten. In this work we use several efficient techniques in each of the three principal phases forming a the system of recognition which are firstly the pre-processing then secondly the features extraction then finally learning-classification several studies has been done for recognition of Handwritten Tifinagh regions recognition by using in the features extraction phase the square and triangular zoning method in one hand or in the learning-classification phase the support vectors machines (SVM) and the neural networks on the other hand.
• Amazigh alphabet is considered as a national language since a new constitution of 2011 is a creative field [3], [4], [5], [6], [7] is very useful to create a system for Tifinagh hand writing words representing the regions.

## Experimental design, materials and methods

**Fig. 1:**
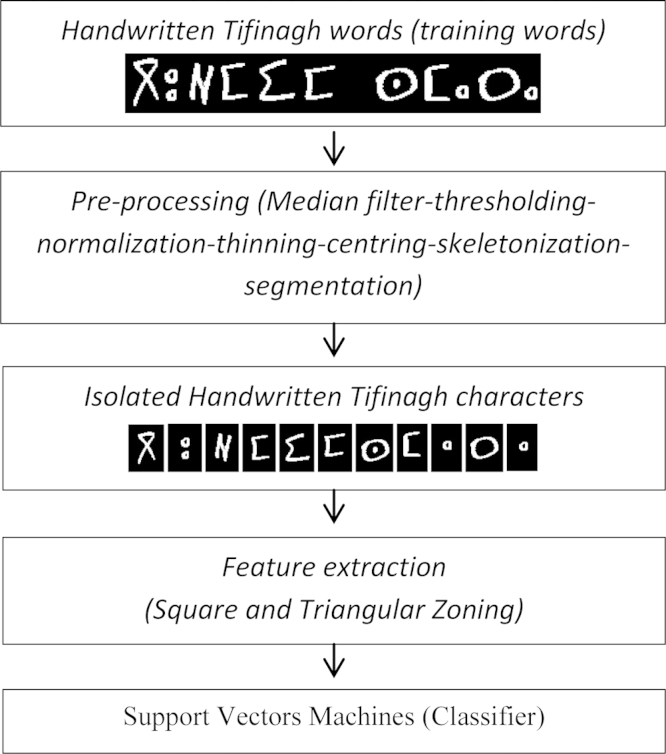
The proposed system for handwritten Tifinagh words recognition.

For several years, on-line and off-line handwriting character recognition has been considered as a very dynamic field given that its applicability in many different domains such as bank check processing, automatic data entry and postal sorting, The postal automation, bank checks identification, automatic processing of administrative files, etc. In this work we have presented the steps of the recognition system in Fig. 1.

We chose a database word contains 1000 words written with marker and that represents sixteen region of Morocco Table 1.

The extraction steps were

**Fig. 2:**
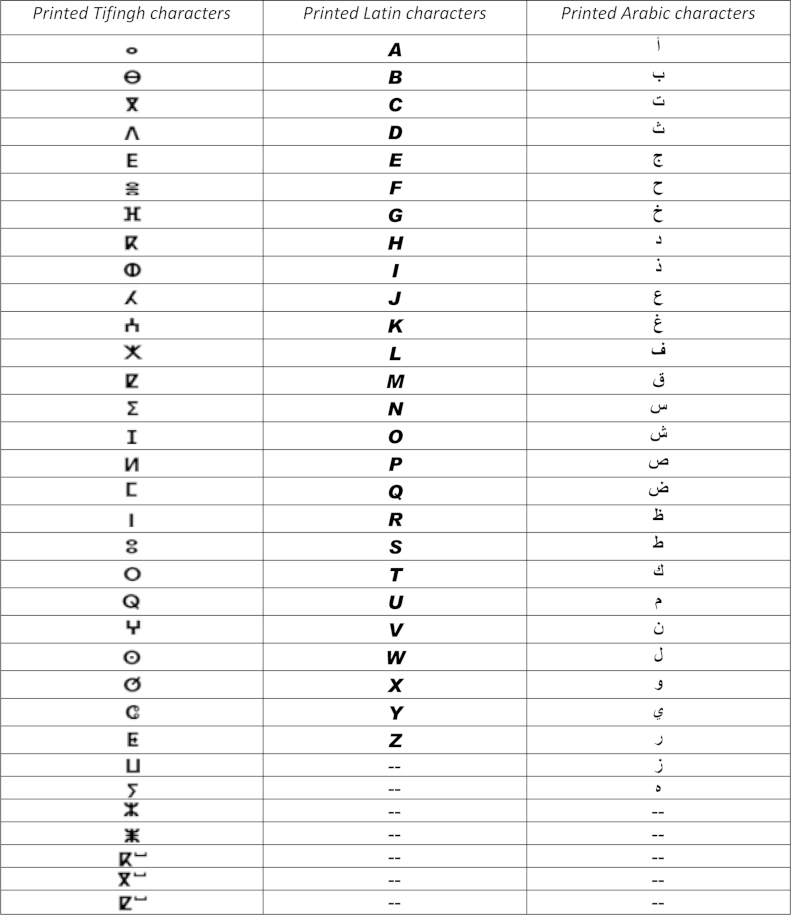
Comparison between the Tifinagh, Arabic and Latin characters.

**Fig. 3:**
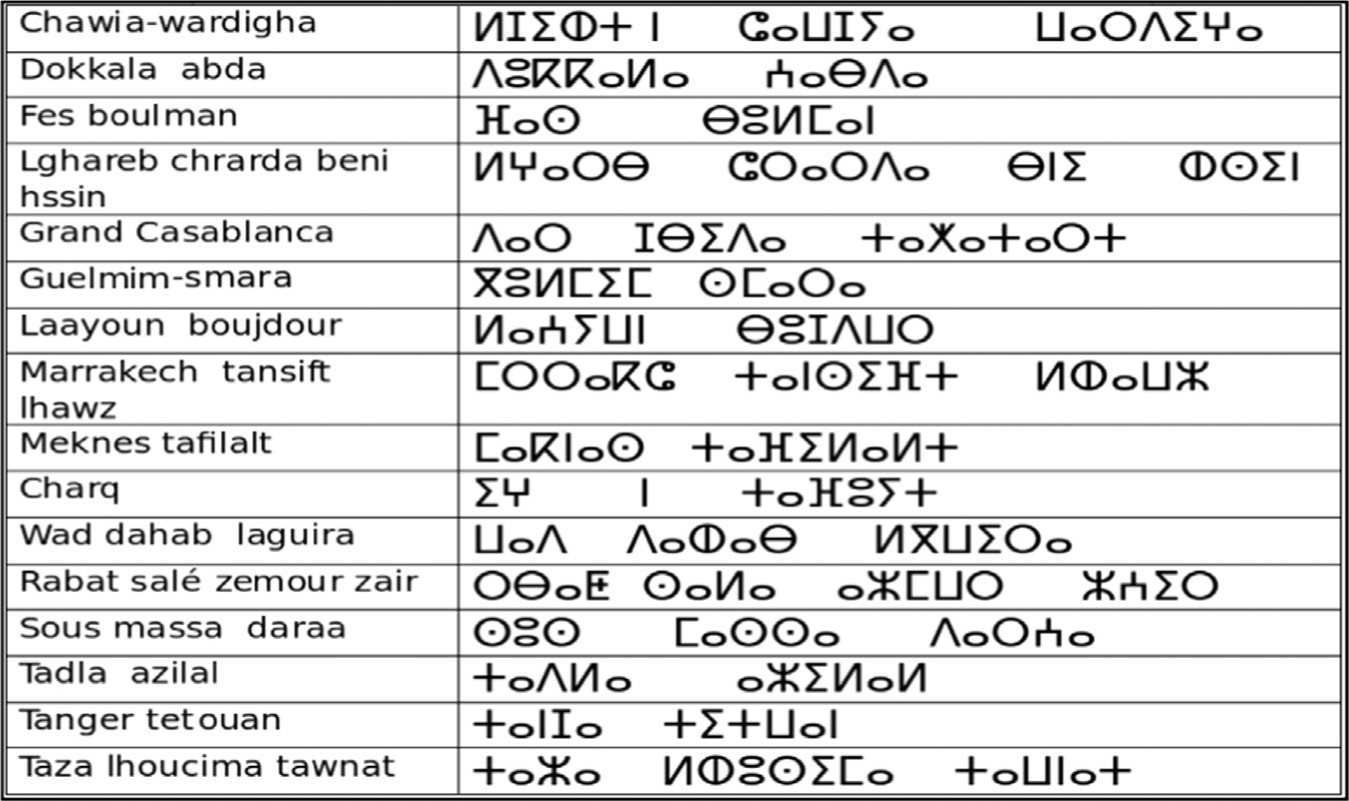
Sixteen regions with Amazigh language.

**Fig. 4:**
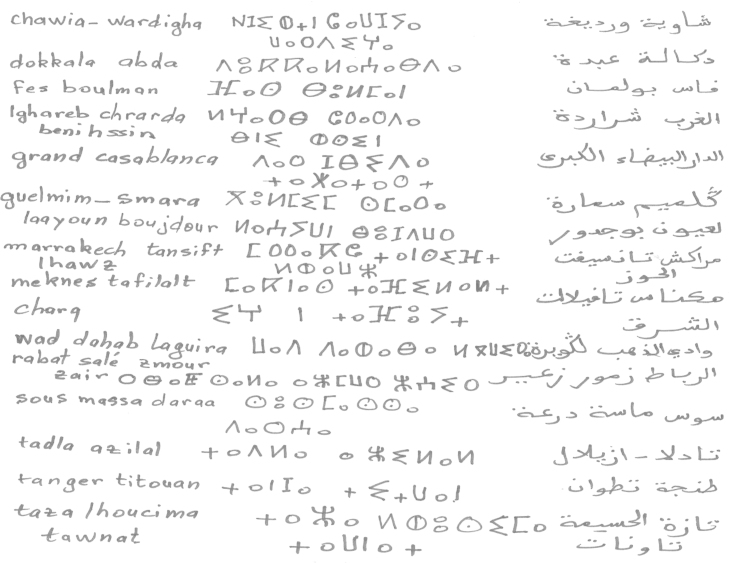
Example of handwritten Tifinagh region from the proposed data base.

**Fig. 5:**
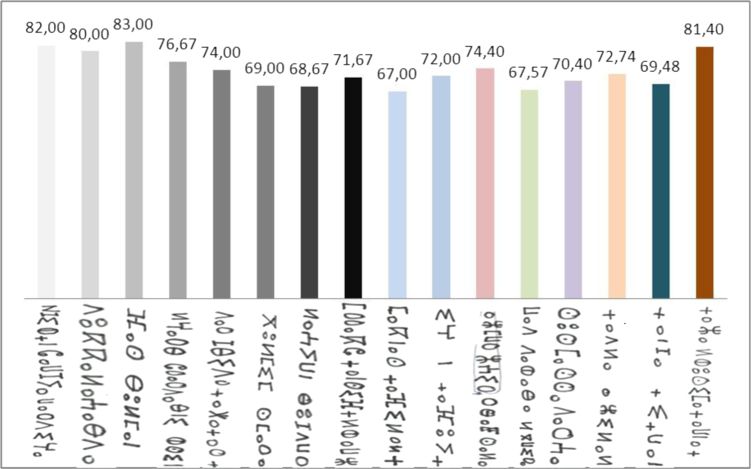
The graphical representation of recognition rate τr for each region.

**Fig. 6:**
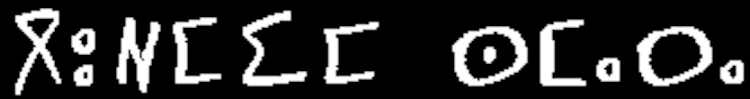
The original image.

**Fig. 7:**
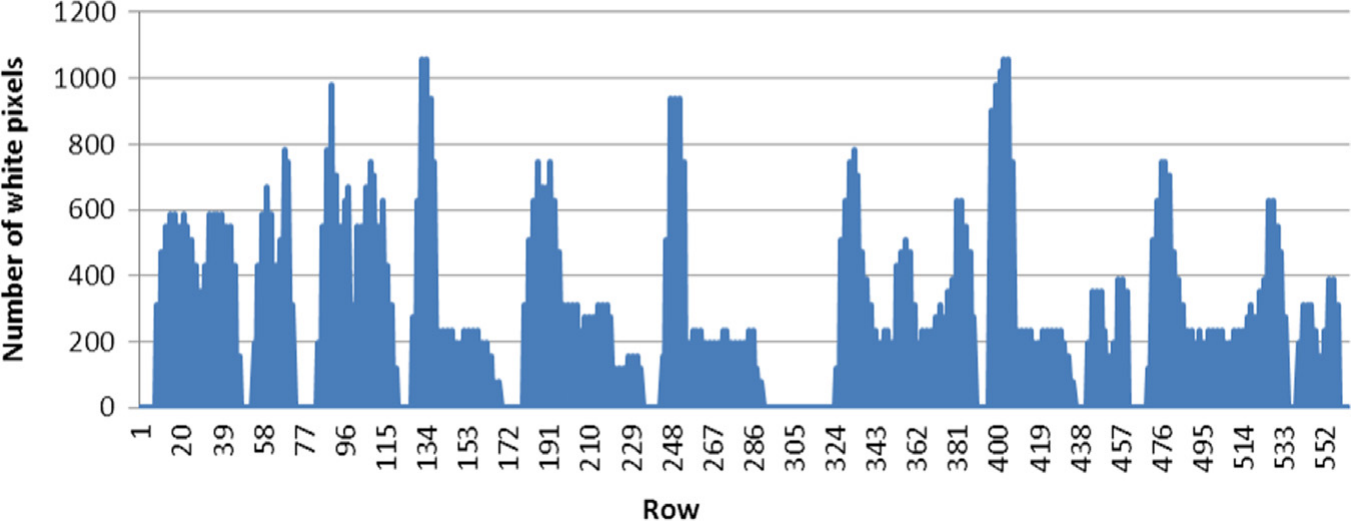
Graphical representation of segmentation column.

**Fig.8:**
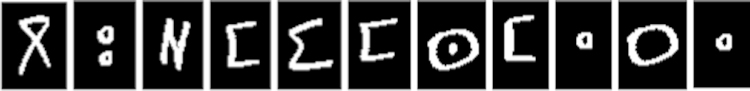
The segmentation in columns.

**Fig. 9:**
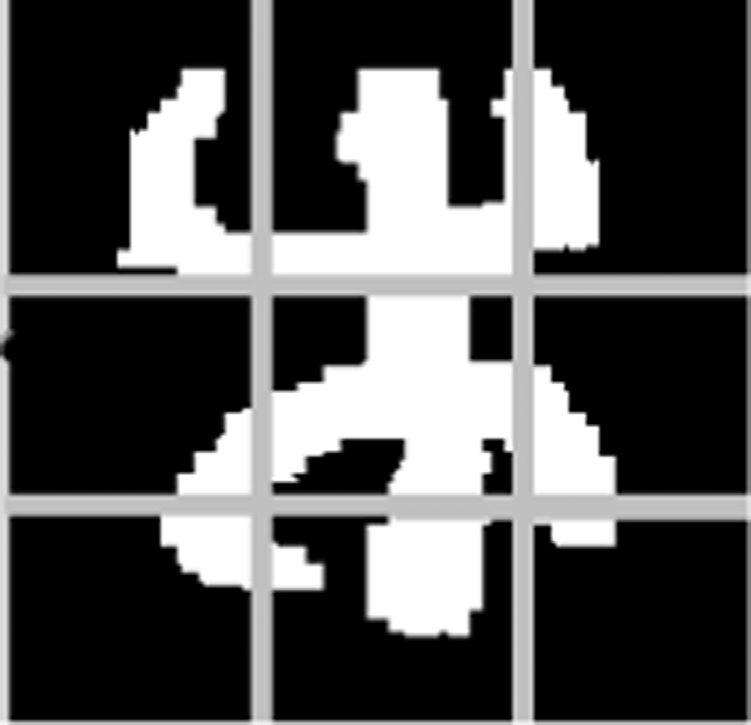
The square zoning method.

**Fig. 10:**
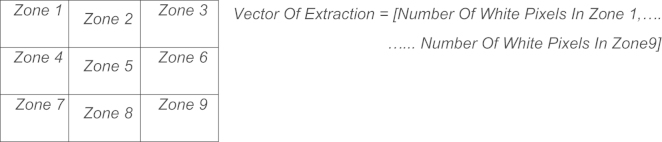
Processes of feature extraction by square zoning.

**Fig. 11:**
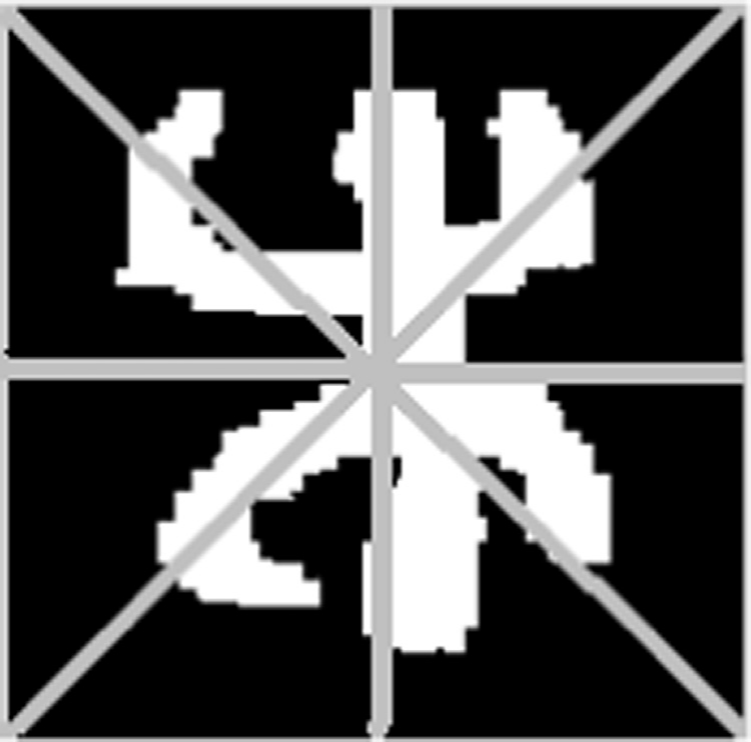
The triangular zoning method.

**Fig. 12:**
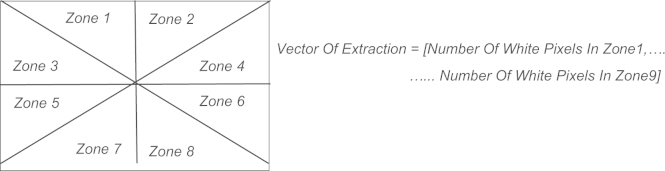
Processes of feature extraction by triangular zoning.

**Fig. 13:**
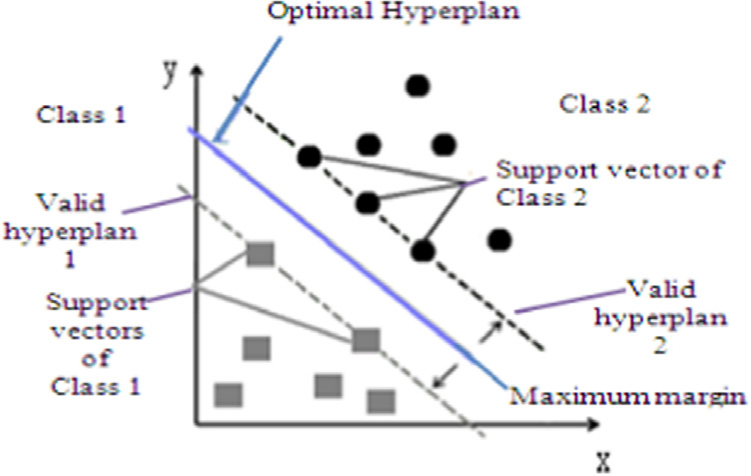
The determination of optimal hyperplane, vectors supports, maximum Marge and valid hyperplanes.

**Fig. 14:**
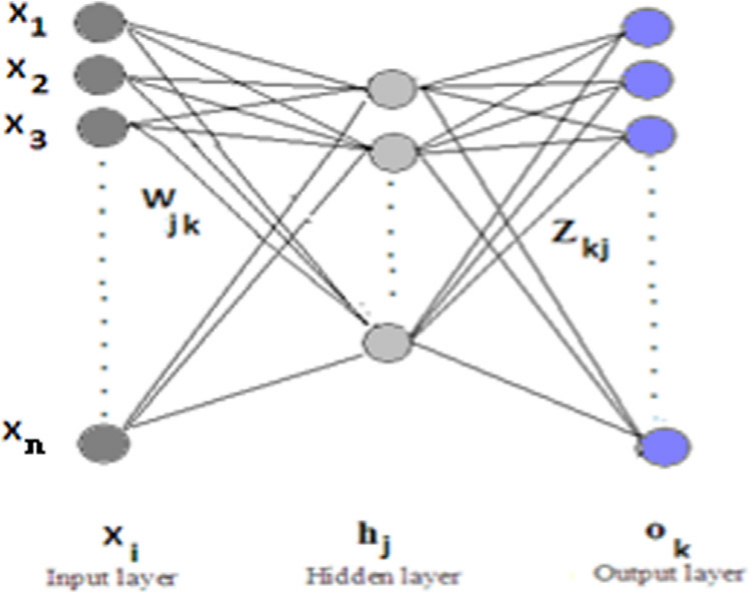
The multi-layer perceptron.

**Fig. 15:**
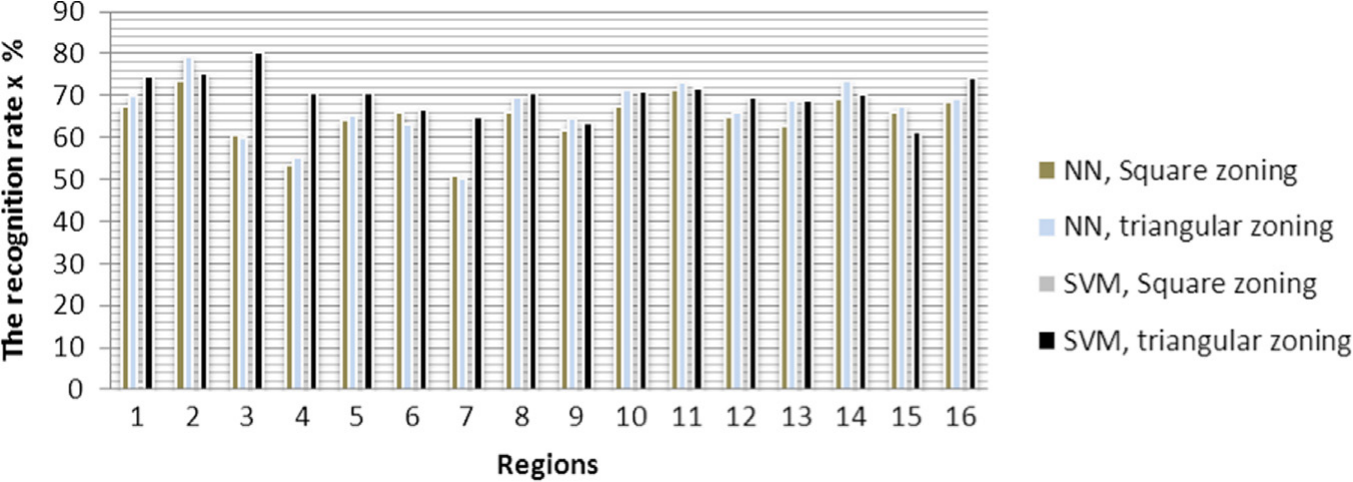
The graphical representation of recognition rate *τ*_r_ of each region with all methods.

**Fig. 16:**
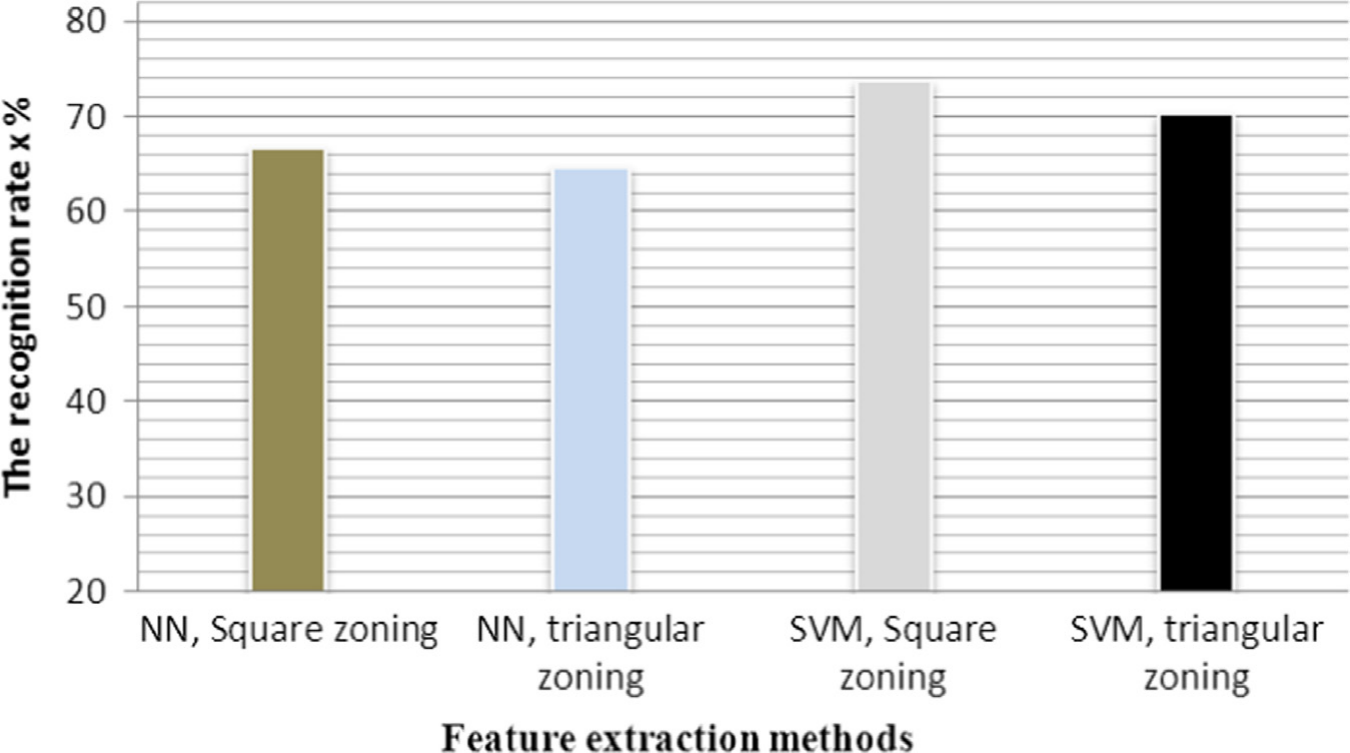
The graphical representation of recognition rate *τ*_r_ of all feature extraction methods.

**Fig. 17:**
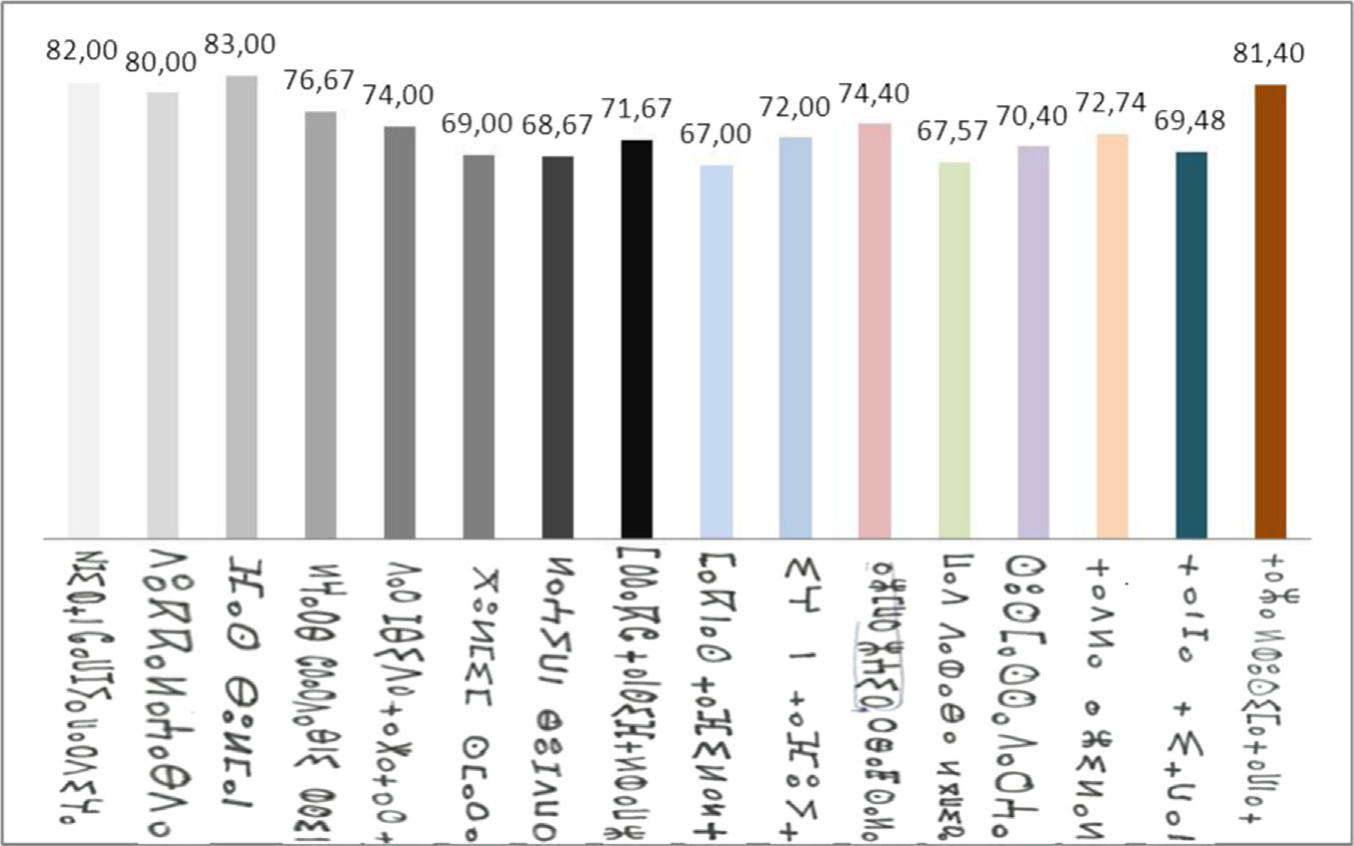
The graphical representation of recognition rate *τ*_r_ of square zoning method and SVM classifier.

• We ask 70 students (in Laboratory of Information Processing and Decision Support) to write the 16 region with Tifinagh characters (Fig. 3).
• The direction of writing of this character is the left to right in horizontal lines.
• The characters are written in a way separated in the text (see Fig. 2, Fig. 3).
• Each original region image has a size equal to 30×30 pixels (Fig. 4, Fig. 5, Fig. 6, Fig. 7, Fig.8, Fig. 9, Fig. 10, Fig. 11, Fig. 12, Fig. 13, Fig. 14, Fig. 15, Fig. 16, Fig. 17).
• The number of the square zones in features extraction equal to 4, 6 and 9 zones.
• The number of the triangles zones in features extraction equal to 4, 6 and 8 zones.
• Each numeral is transformed to a vector of 4, 6 and 9 components for square zoning and to a vector of 4, 6 and 8 components of triangular zoning in features extraction.
• The standard deviation of the GRBF kernel function is equal to 0.1 in classification phase with support vectors machines.
• The degree of the Polynomial (POL) kernel function is equal to 10 and their parameters *a*=*b*=1 in classification phase with support vectors machines.
• We realized a variation on the size of the zones in features extraction to find the best performing method.
• To do this, we have chosen the values {5, 10, 15} of hidden layer neurons number.

The graphical representation to recognition rate of each region *τ*_r_ is shown in Fig. 5.

## Figures and Tables

**Table 1:** The obtained recognition rates *τ*_r_ and *τ*_g_ by each hybrid method and each classifier.

**Regions**	**Neural networks**		**Support vectors machines**	
**Square zoning**	**Triangular zoning**	**Square zoning**	**Triangular zoning**
	70.00	67.57	82.00	74.49
	79.13	73.4	80.00	75.18
	60.00	60.74	83.00	80.34
	55.09	53.48	76.67	70.61
	65.21	64.00	74.00	70.78
	63.25	65.85	69.00	66.60
	50.18	50.97	68.67	65.00
	69.66	65.93	71.67	70.56
	64.46	61.71	67.00	63.60
	71.31	67.40	72.00	70.96
	73.00	71.43	74.00	71.73
	66.11	64.84	67.57	69.41
	68.67	62.69	70.40	69.00
	73.37	69.29	72.74	70.44
	67.45	66.04	69.48	61.33
	69.26	68.34	81.4	74.18
*τ*_*g*_	66.64	64.61	73.73	70.26

All values of the recognition rate for each region *τ*_r_ (given in %) and also those of the global rate recognition *τ*_*g*_ of all 16 regions (given in %) which we have obtained in the table.
