# Supplementary figures and images for: Handwriting Moroccan regions recognition using Tifinagh character
Source: Data Brief. 2015 Jul 26;4:534–43. doi: 10.1016/j.dib.2015.07.018 (PMC4783523; doi:10.1016/j.dib.2015.07.018)

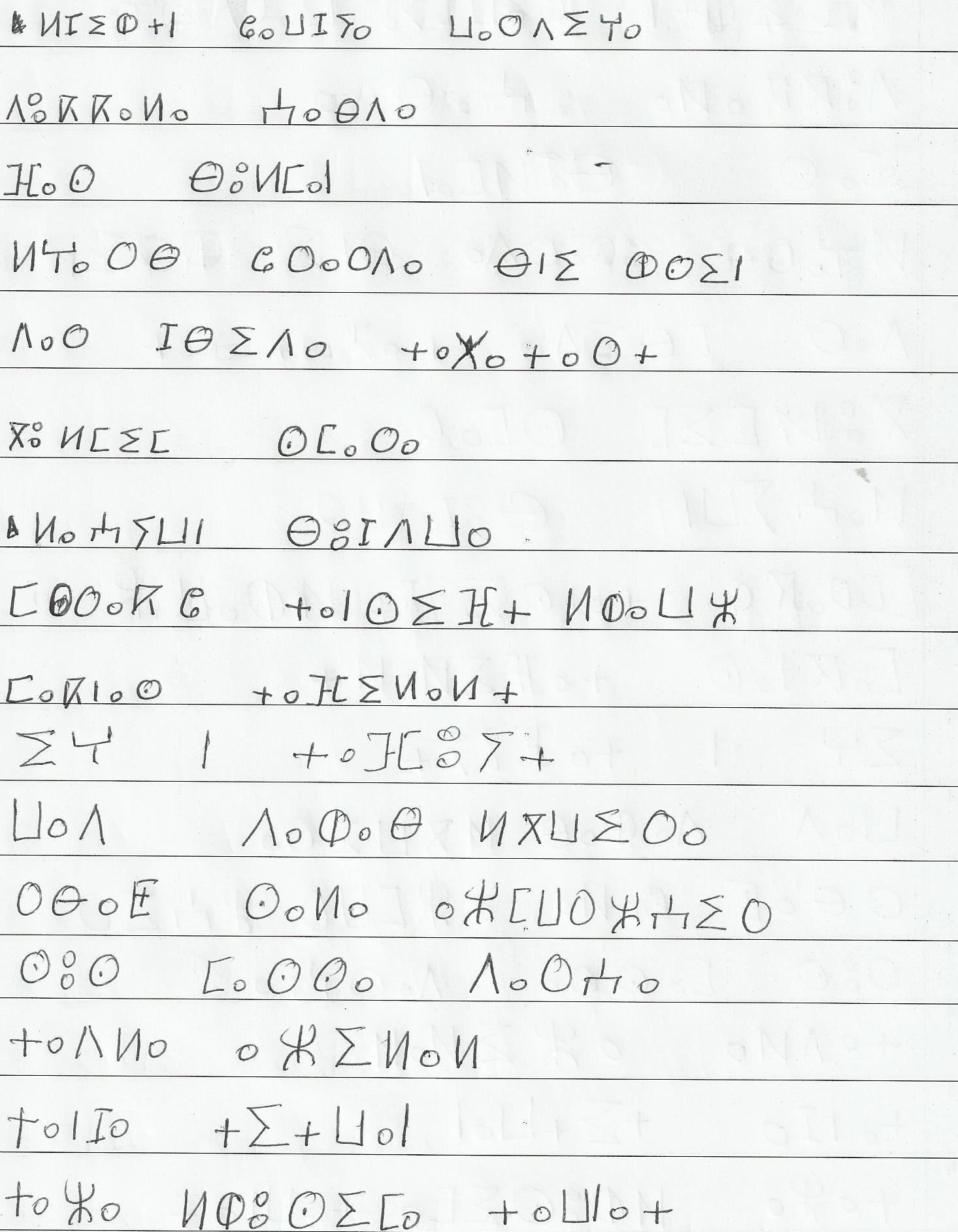

Supplement: Supplementary file 1 — Supplementary data [file mmc1.zip › EXAMPLE OF DATABASE/1.jpg]

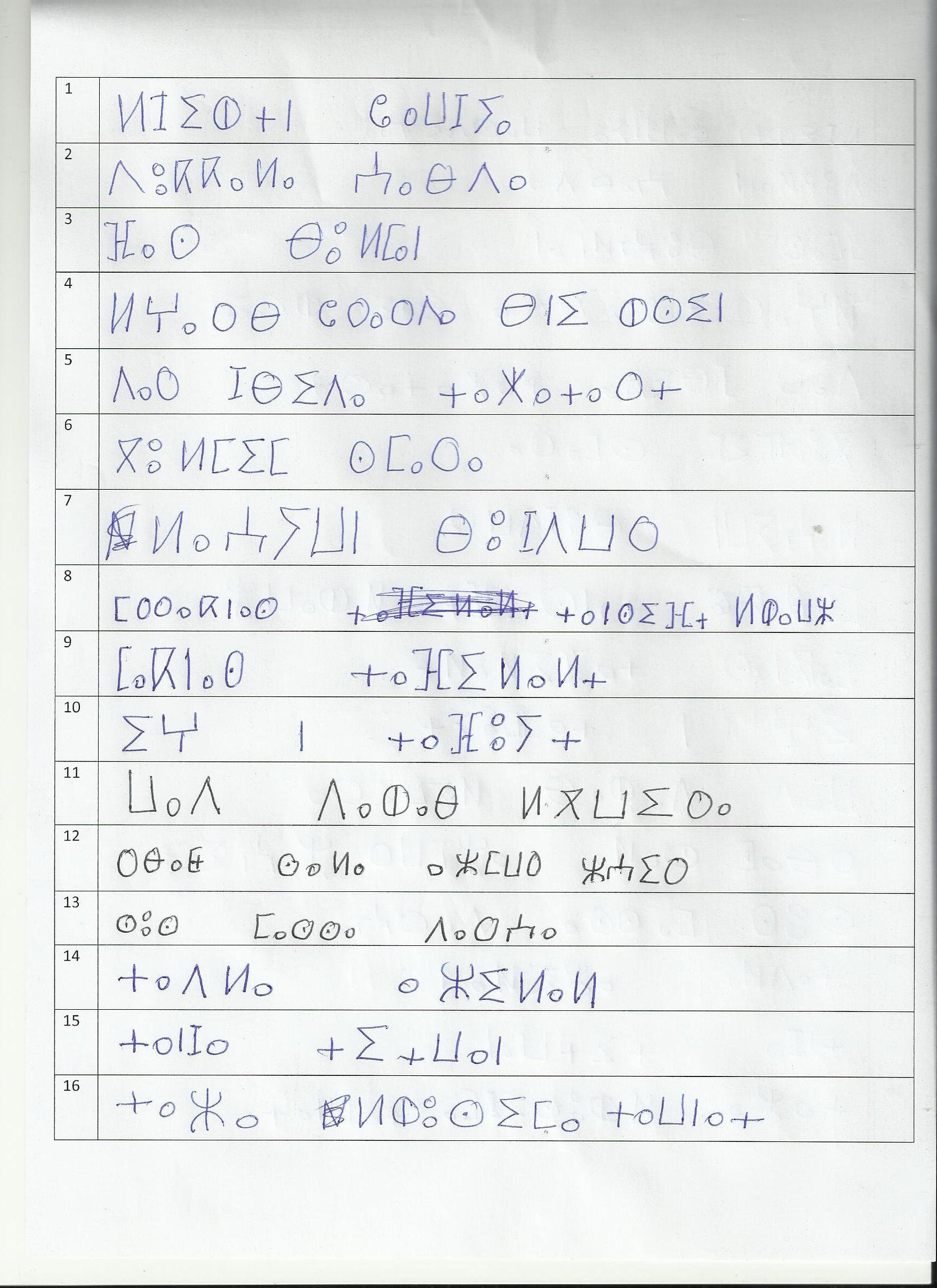

Supplement: Supplementary file 1 — Supplementary data [file mmc1.zip › EXAMPLE OF DATABASE/10.jpg]

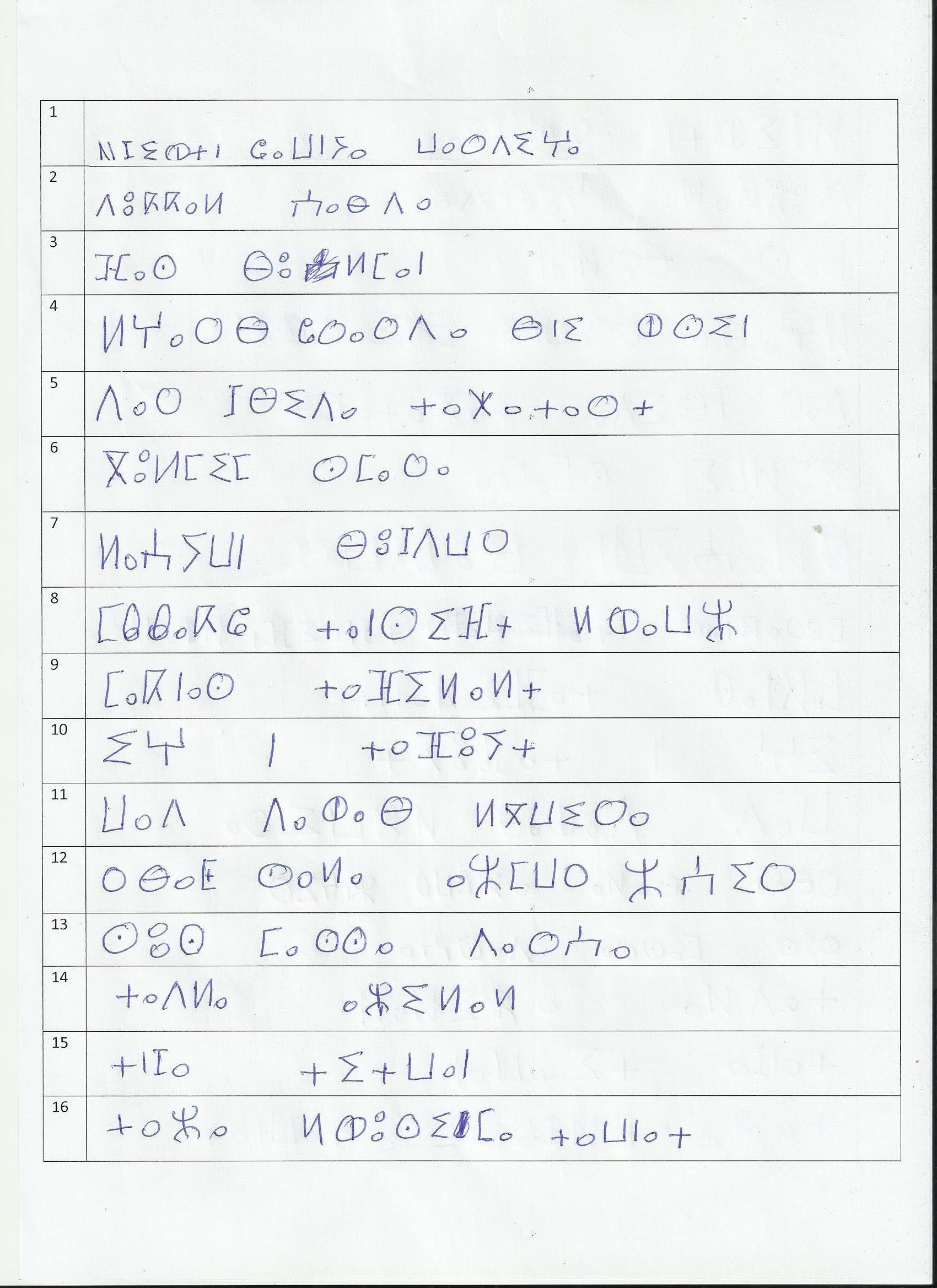

Supplement: Supplementary file 1 — Supplementary data [file mmc1.zip › EXAMPLE OF DATABASE/11.jpg]

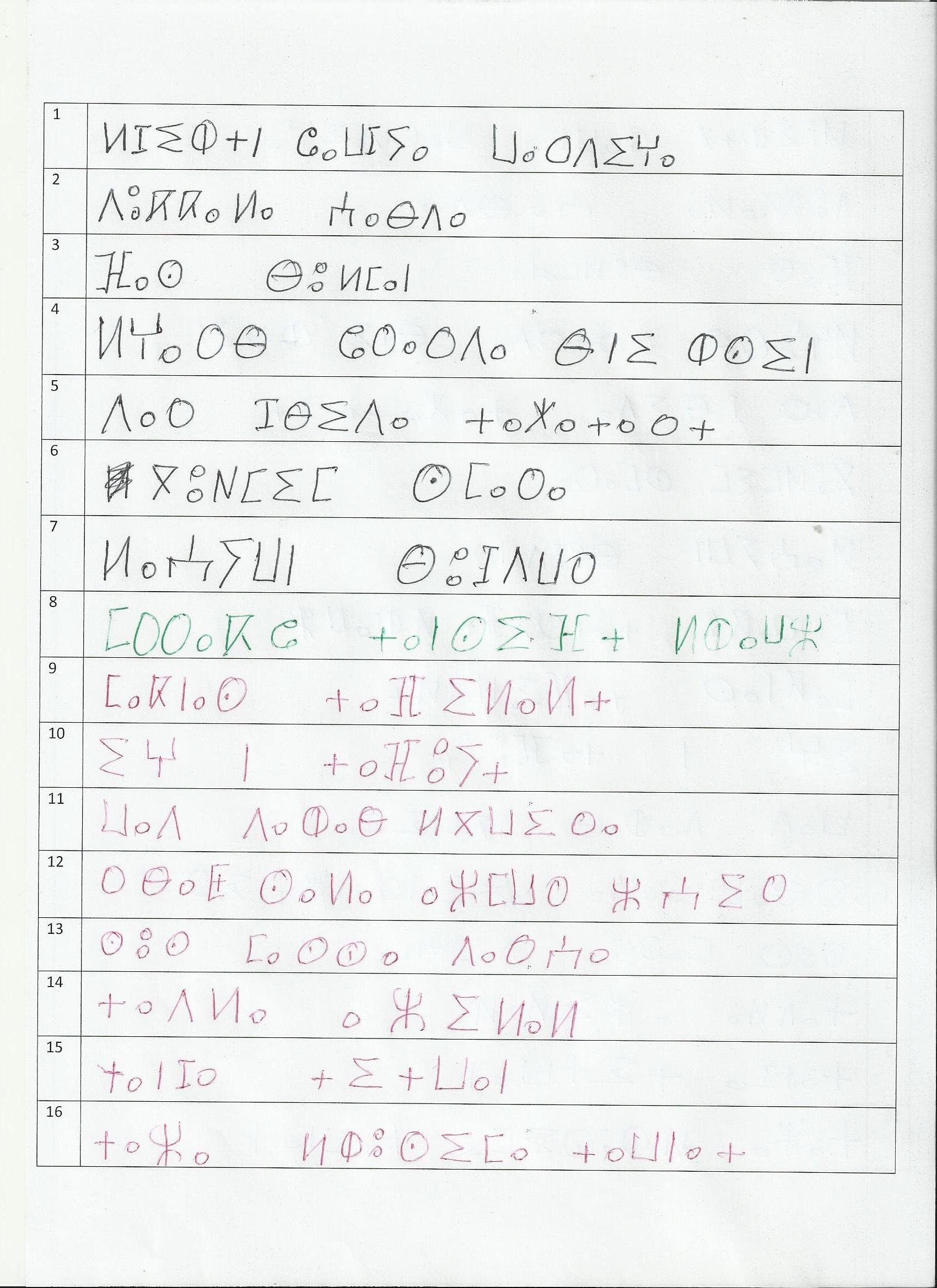

Supplement: Supplementary file 1 — Supplementary data [file mmc1.zip › EXAMPLE OF DATABASE/12.jpg]

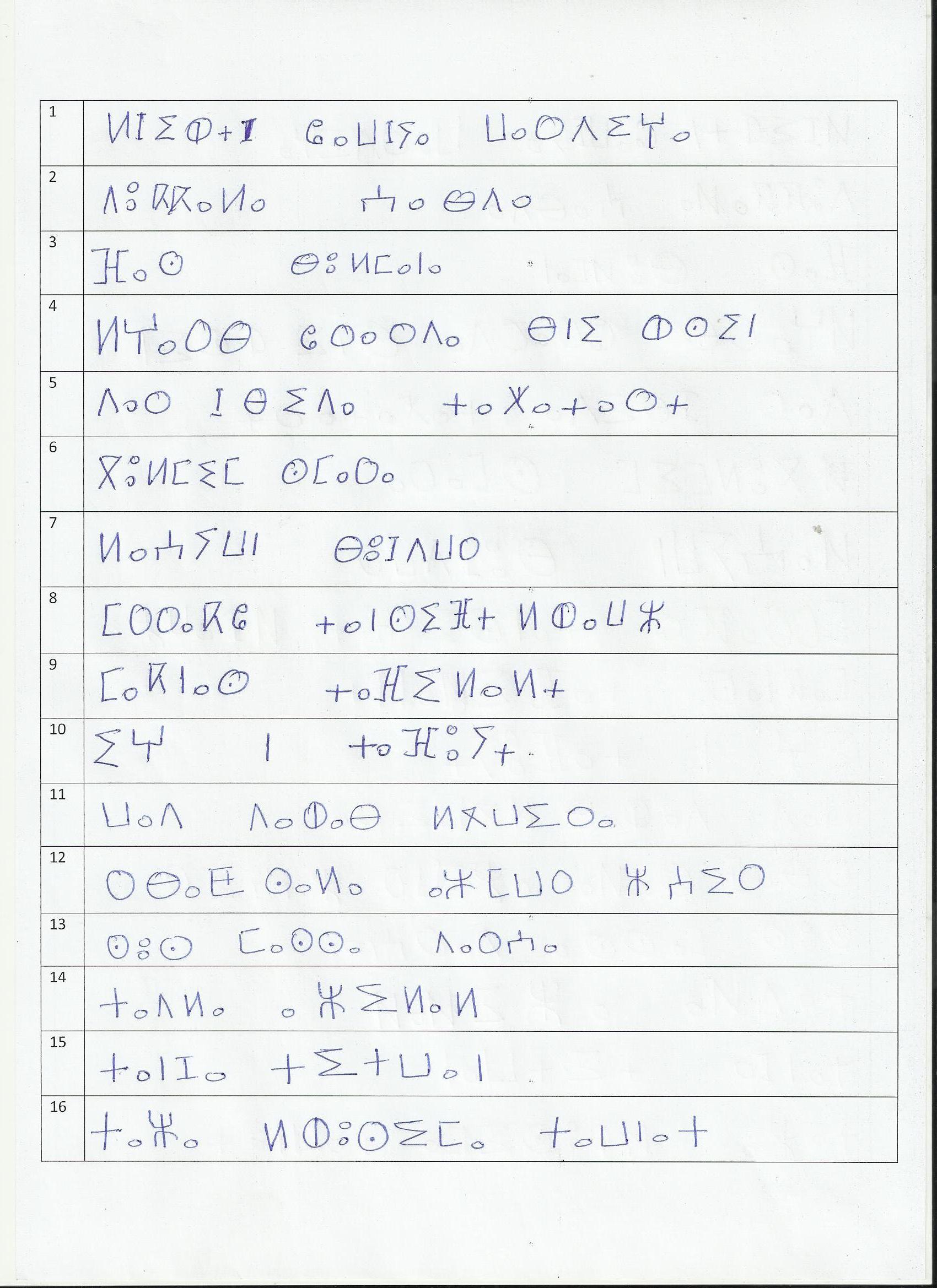

Supplement: Supplementary file 1 — Supplementary data [file mmc1.zip › EXAMPLE OF DATABASE/13.jpg]

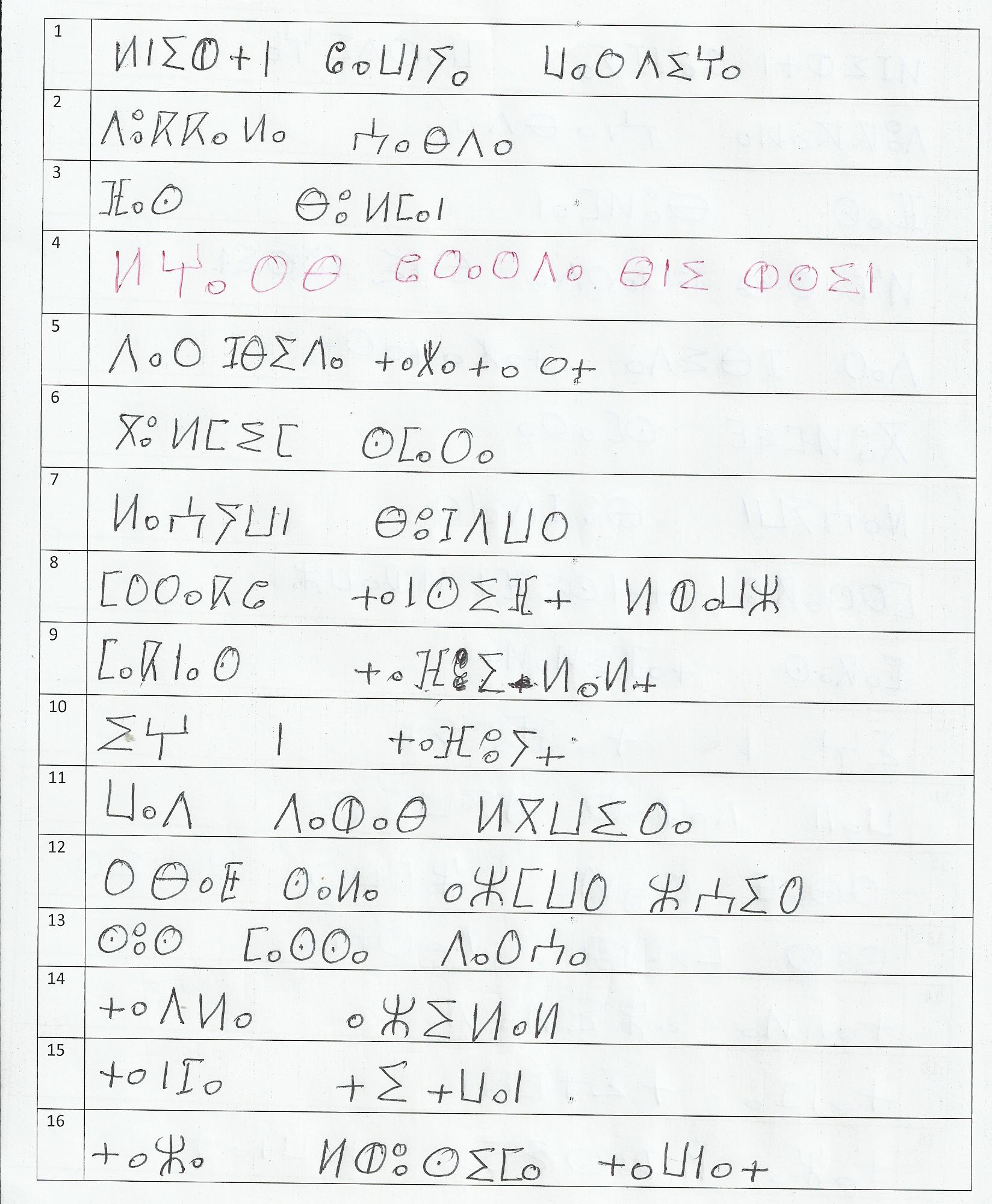

Supplement: Supplementary file 1 — Supplementary data [file mmc1.zip › EXAMPLE OF DATABASE/14.jpg]

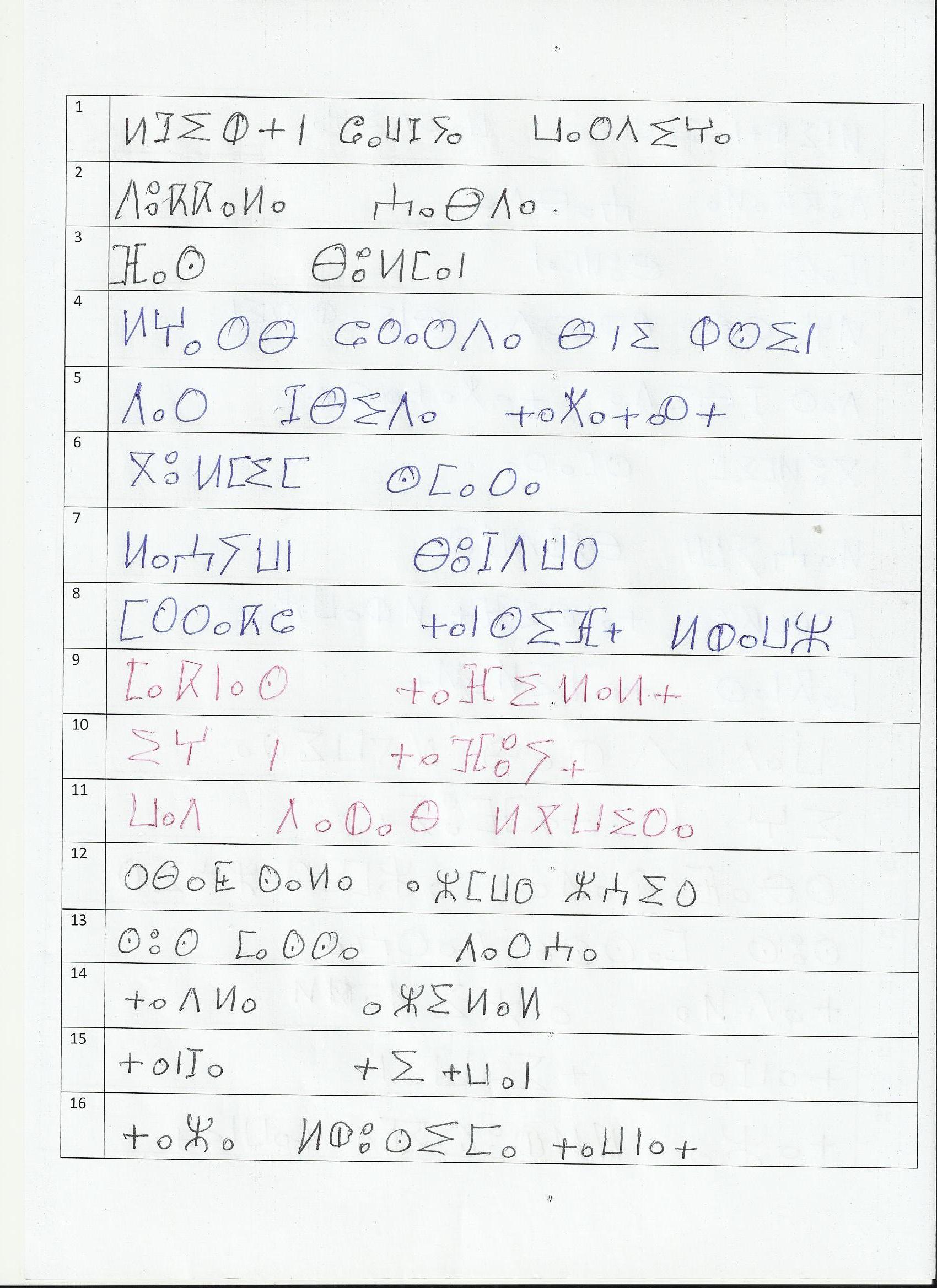

Supplement: Supplementary file 1 — Supplementary data [file mmc1.zip › EXAMPLE OF DATABASE/15.jpg]

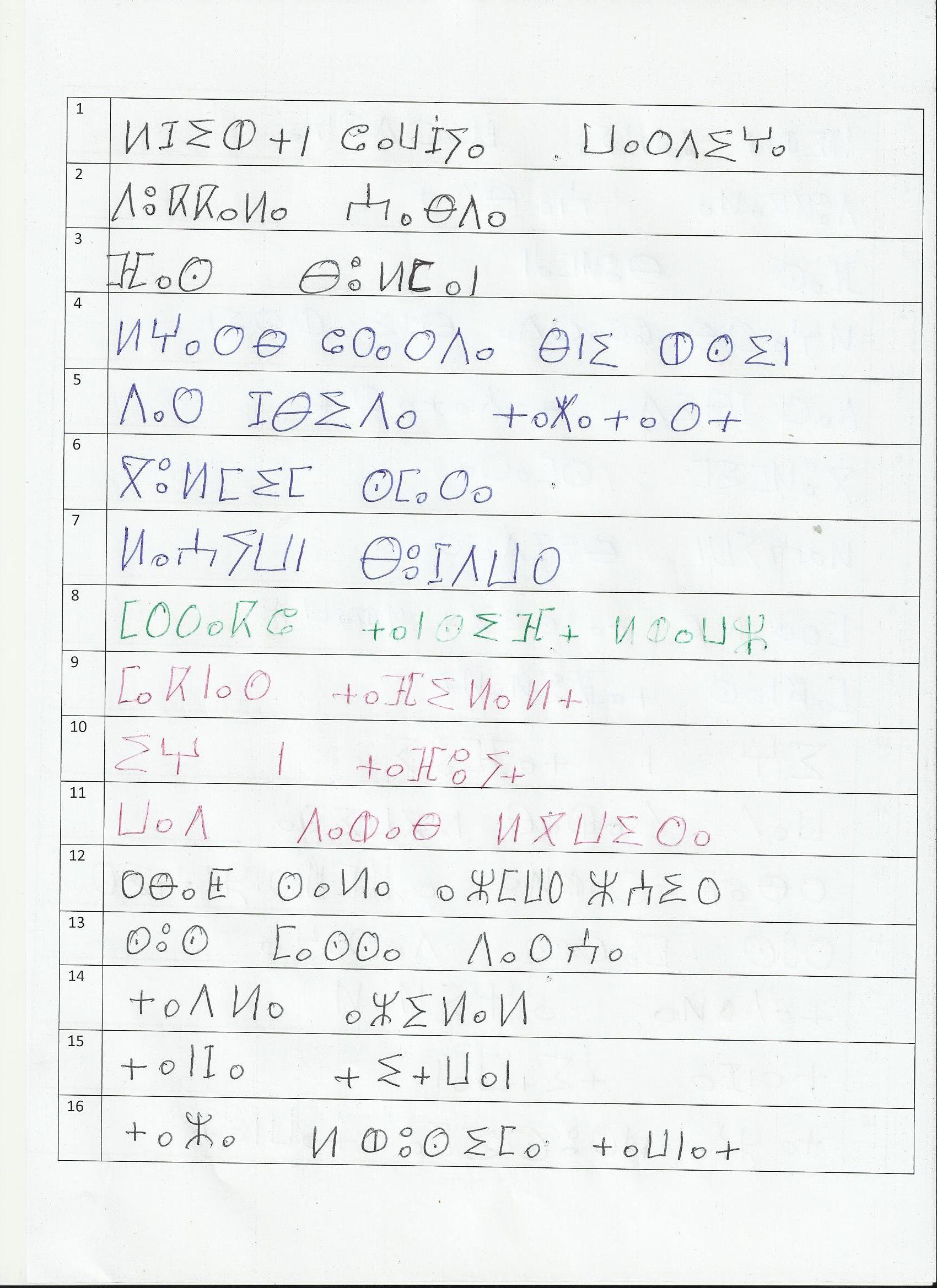

Supplement: Supplementary file 1 — Supplementary data [file mmc1.zip › EXAMPLE OF DATABASE/16.jpg]

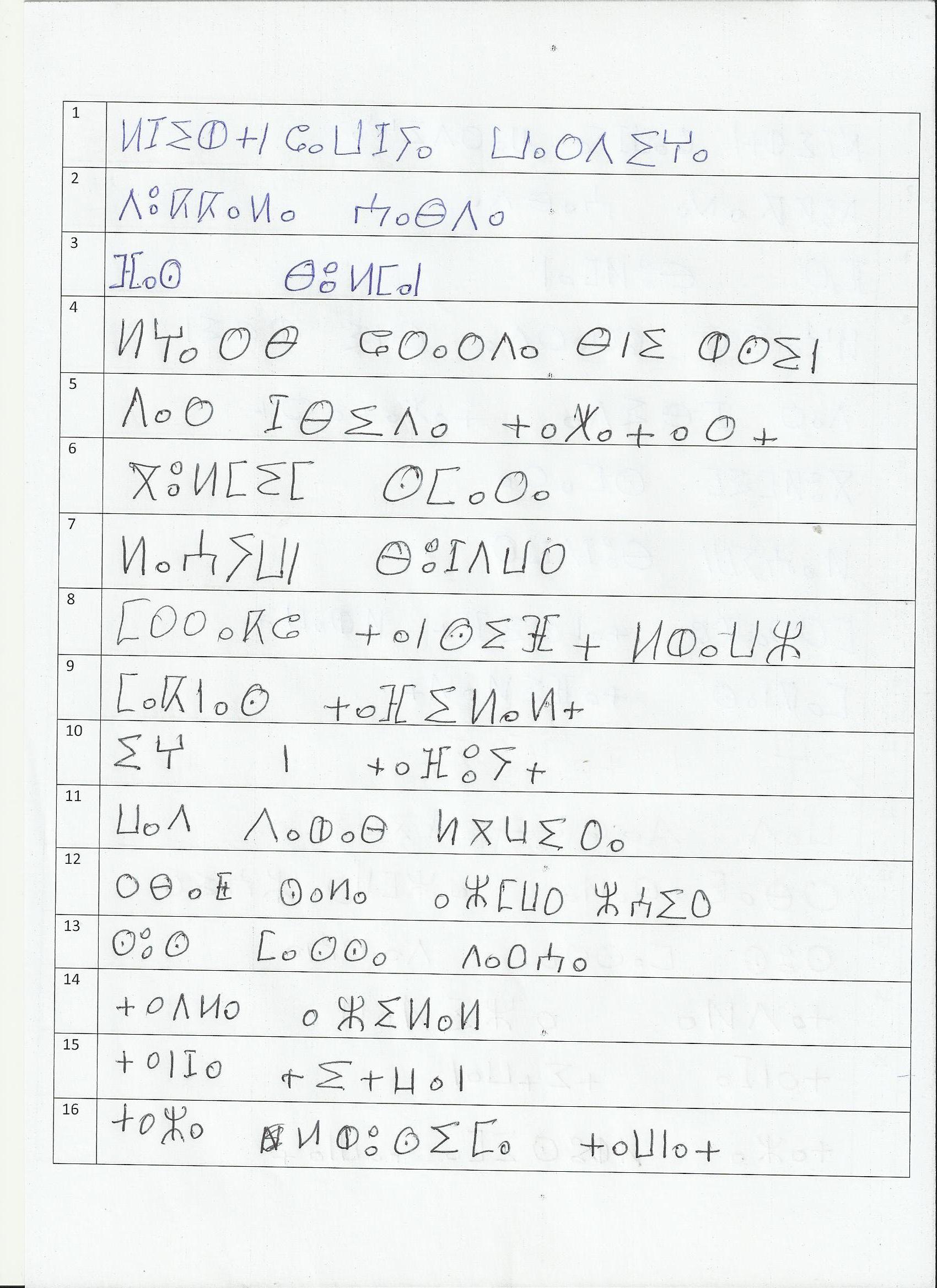

Supplement: Supplementary file 1 — Supplementary data [file mmc1.zip › EXAMPLE OF DATABASE/17.jpg]

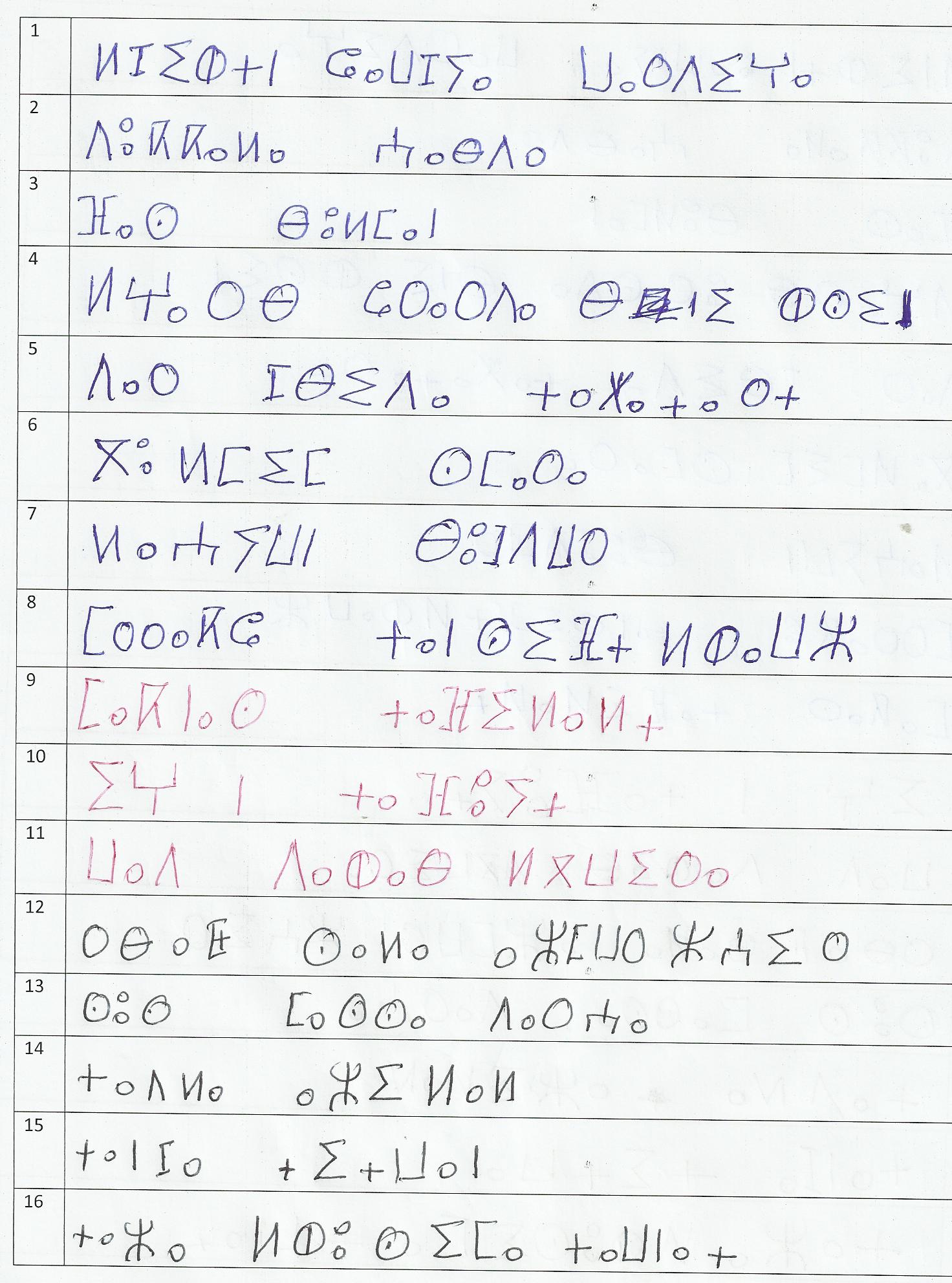

Supplement: Supplementary file 1 — Supplementary data [file mmc1.zip › EXAMPLE OF DATABASE/18.jpg]

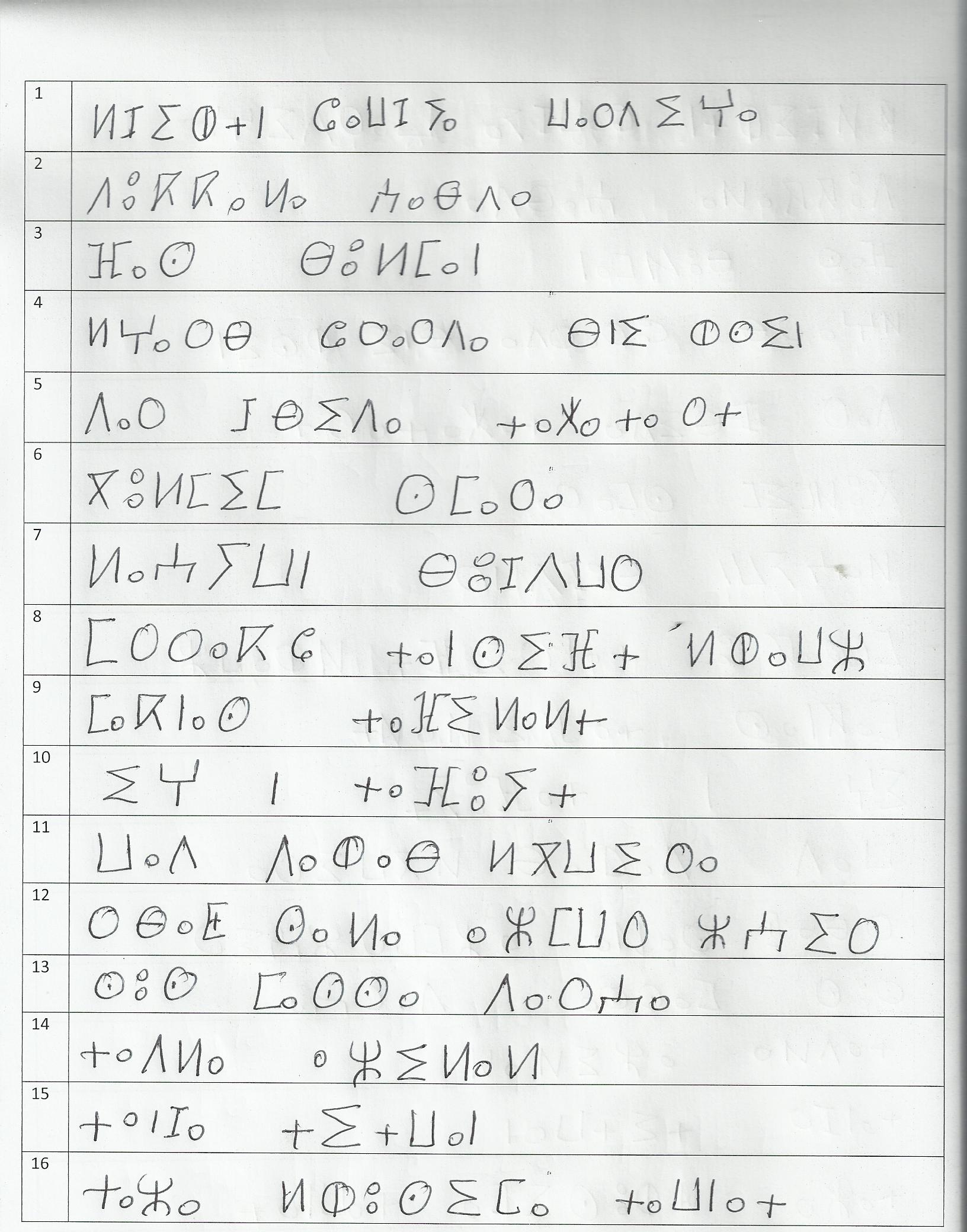

Supplement: Supplementary file 1 — Supplementary data [file mmc1.zip › EXAMPLE OF DATABASE/19.jpg]

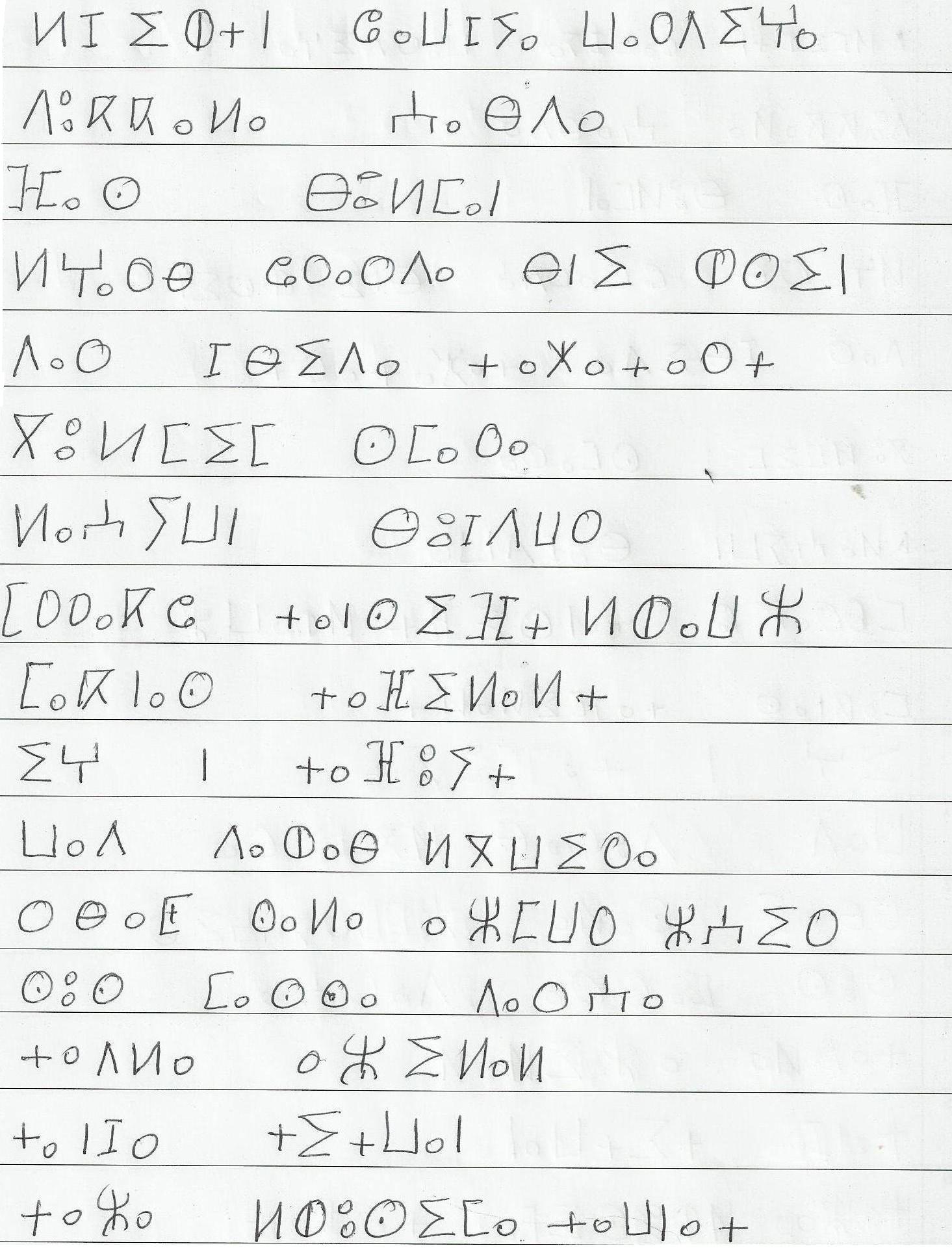

Supplement: Supplementary file 1 — Supplementary data [file mmc1.zip › EXAMPLE OF DATABASE/2.jpg]

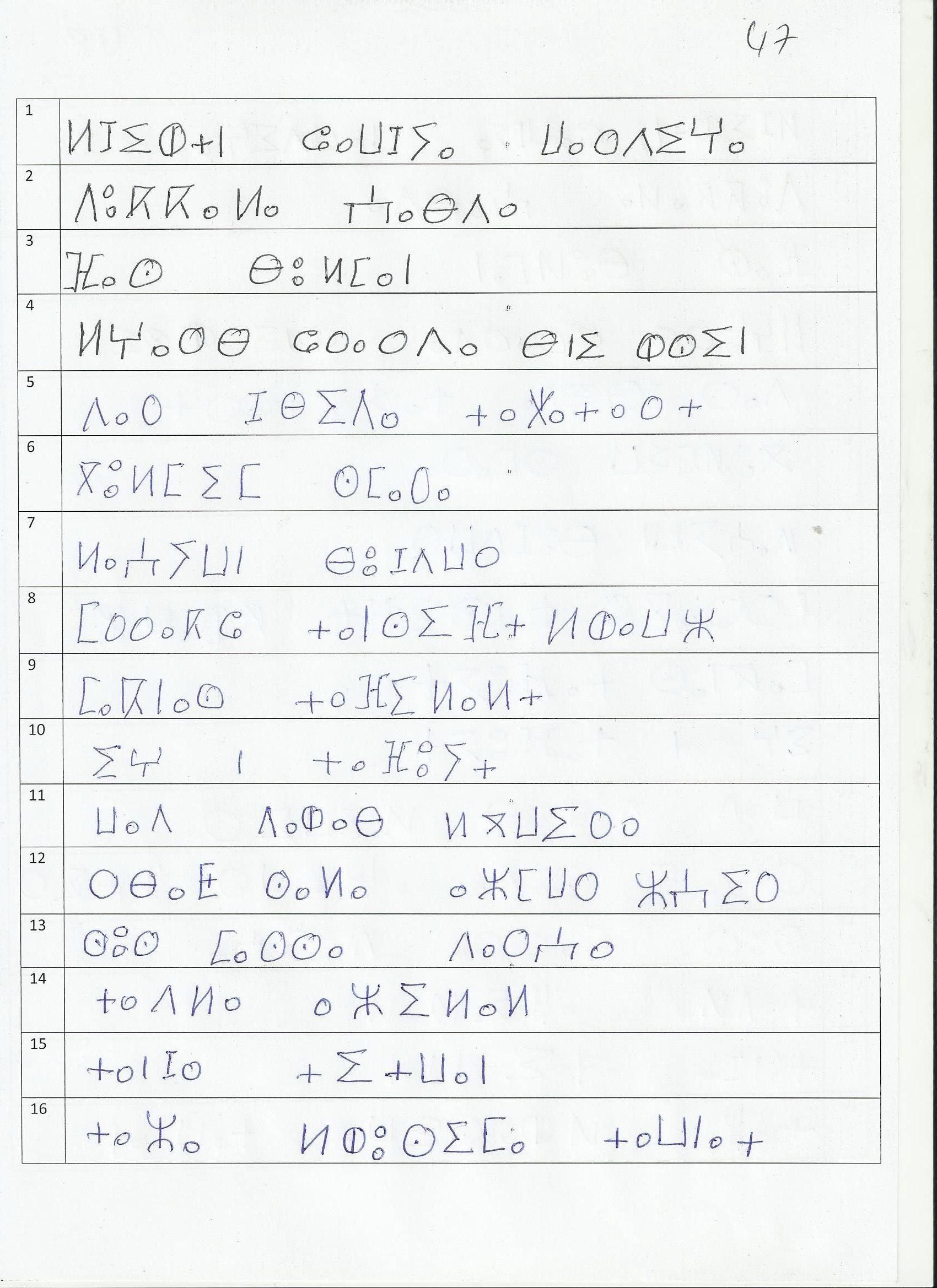

Supplement: Supplementary file 1 — Supplementary data [file mmc1.zip › EXAMPLE OF DATABASE/20.jpg]

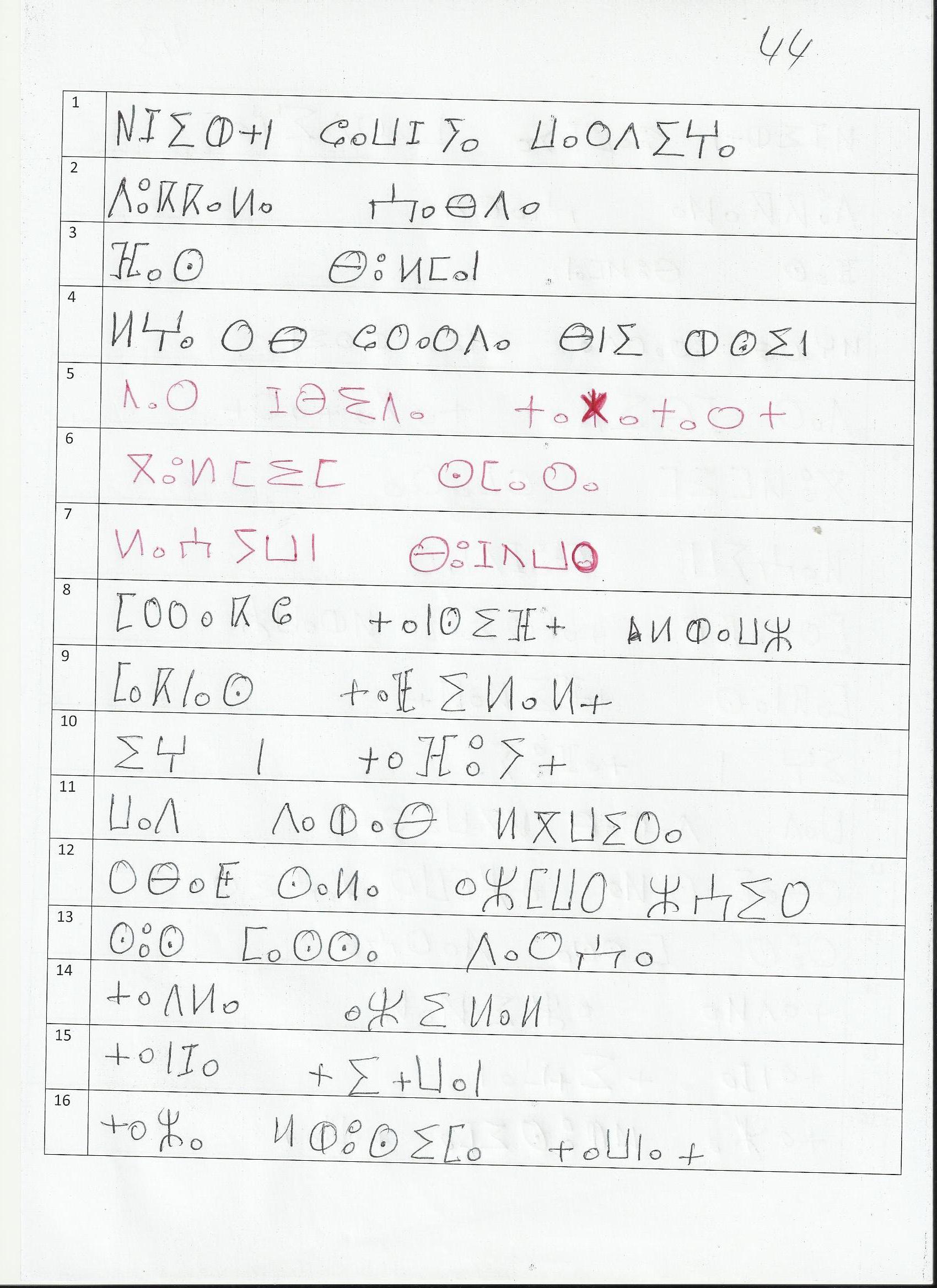

Supplement: Supplementary file 1 — Supplementary data [file mmc1.zip › EXAMPLE OF DATABASE/21.jpg]

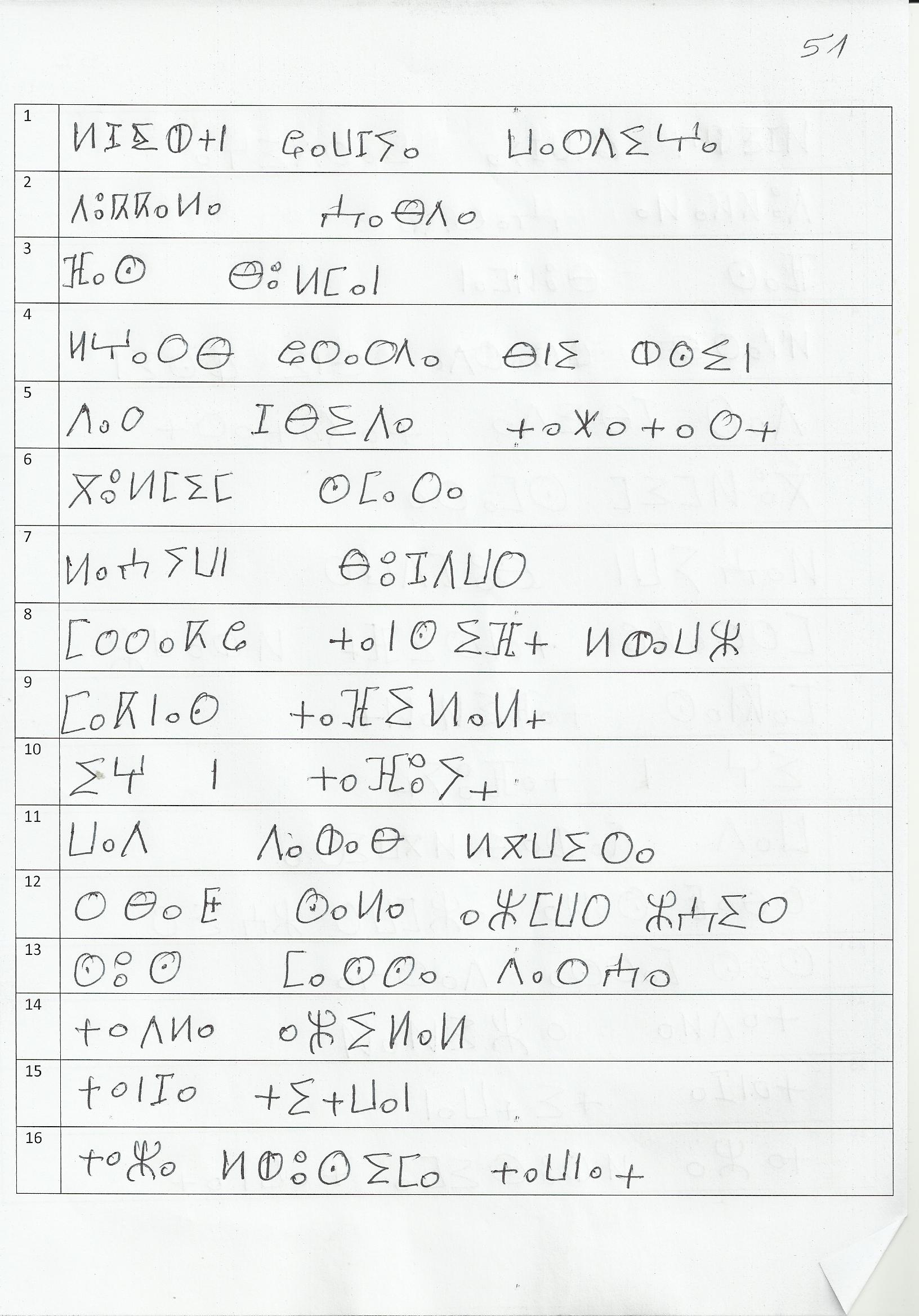

Supplement: Supplementary file 1 — Supplementary data [file mmc1.zip › EXAMPLE OF DATABASE/22.jpg]

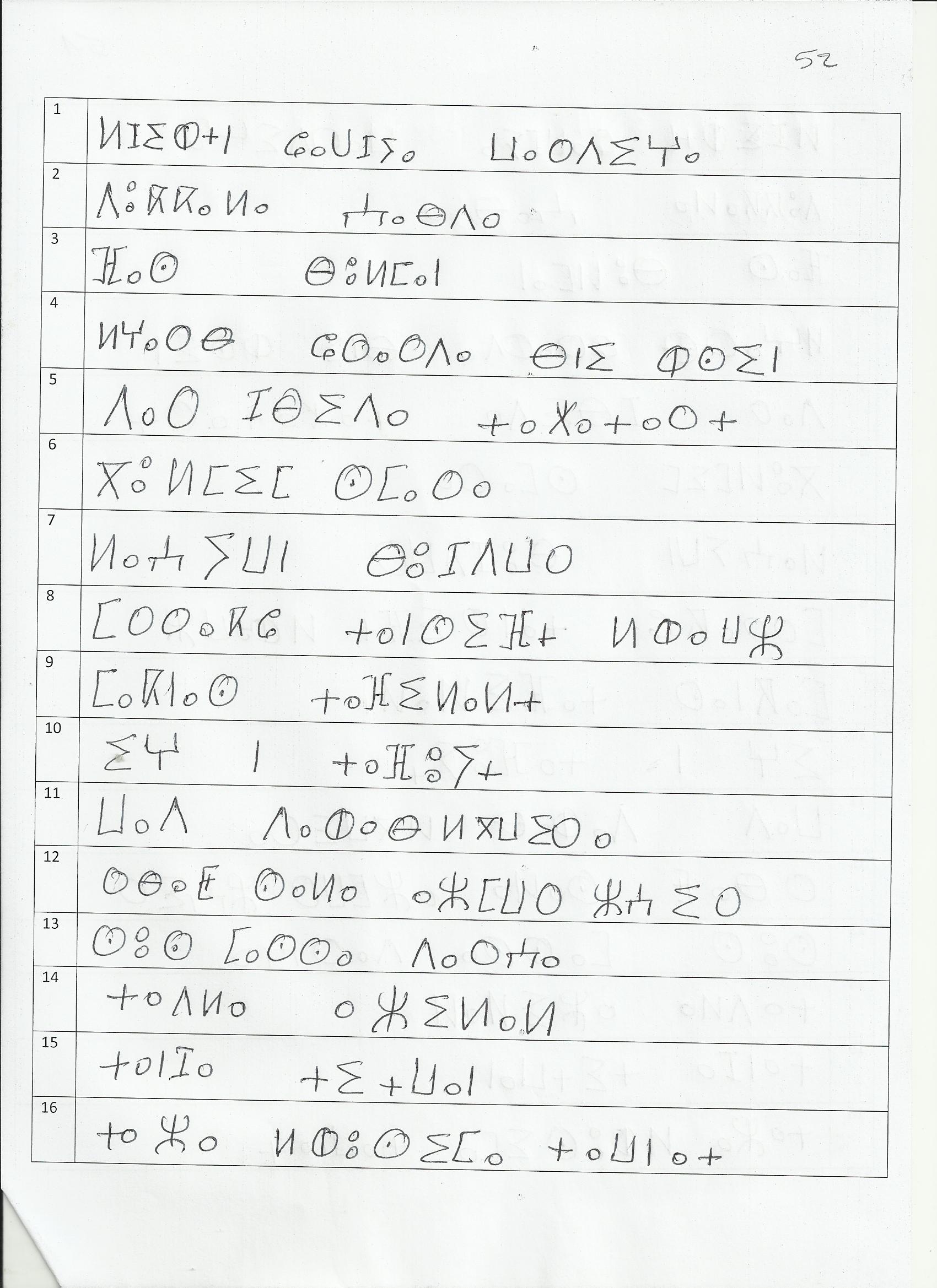

Supplement: Supplementary file 1 — Supplementary data [file mmc1.zip › EXAMPLE OF DATABASE/23.jpg]

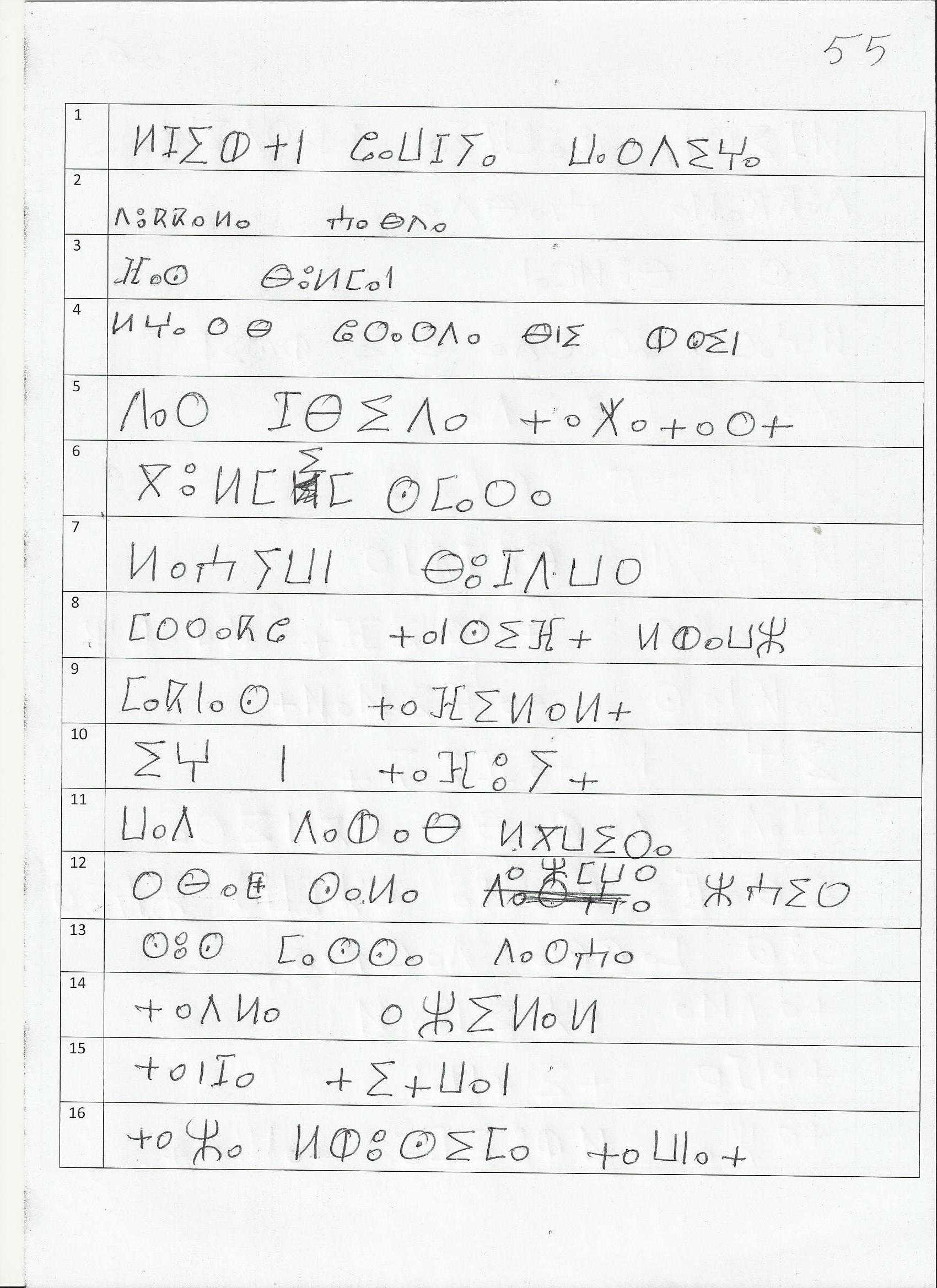

Supplement: Supplementary file 1 — Supplementary data [file mmc1.zip › EXAMPLE OF DATABASE/24.jpg]

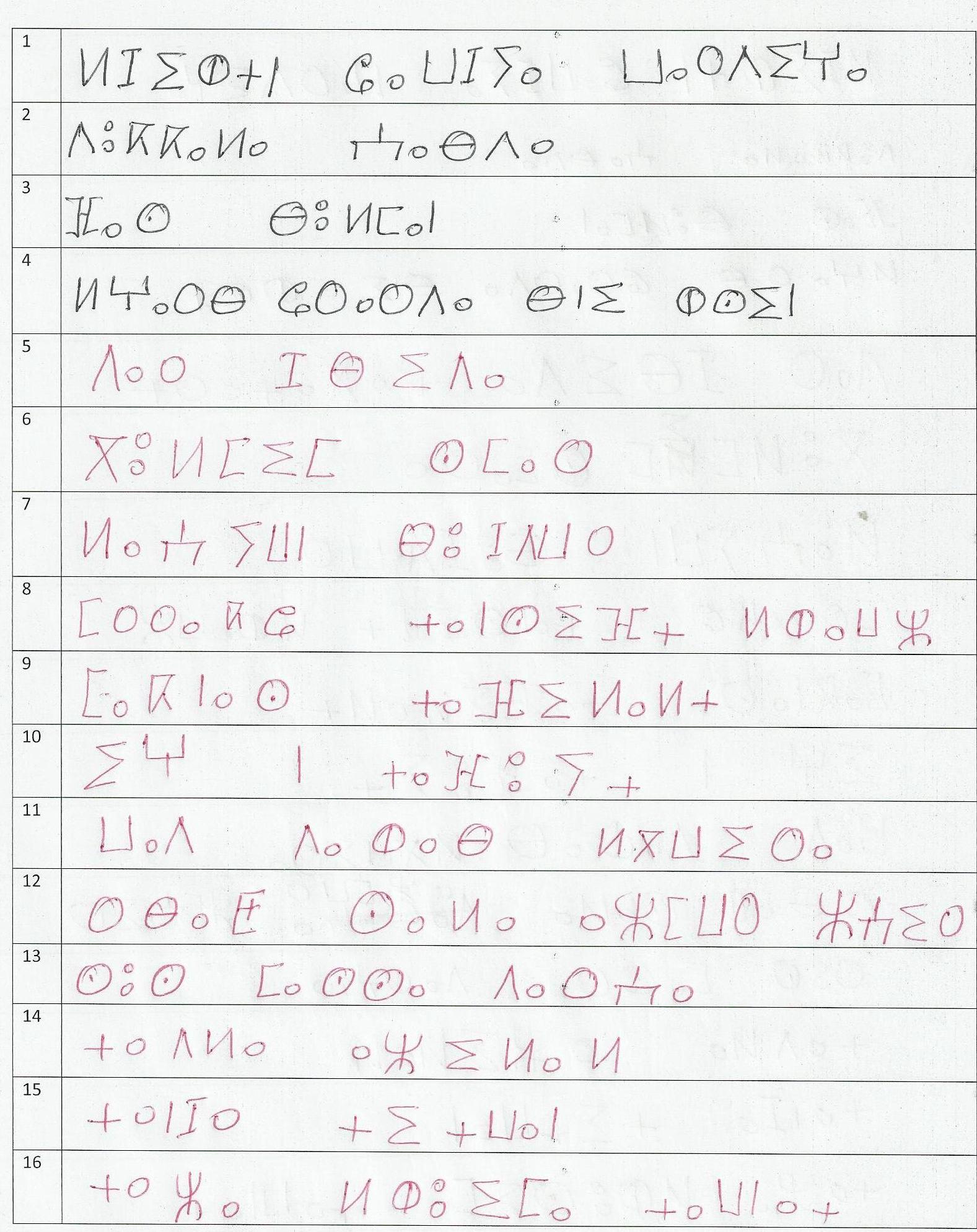

Supplement: Supplementary file 1 — Supplementary data [file mmc1.zip › EXAMPLE OF DATABASE/25.jpg]

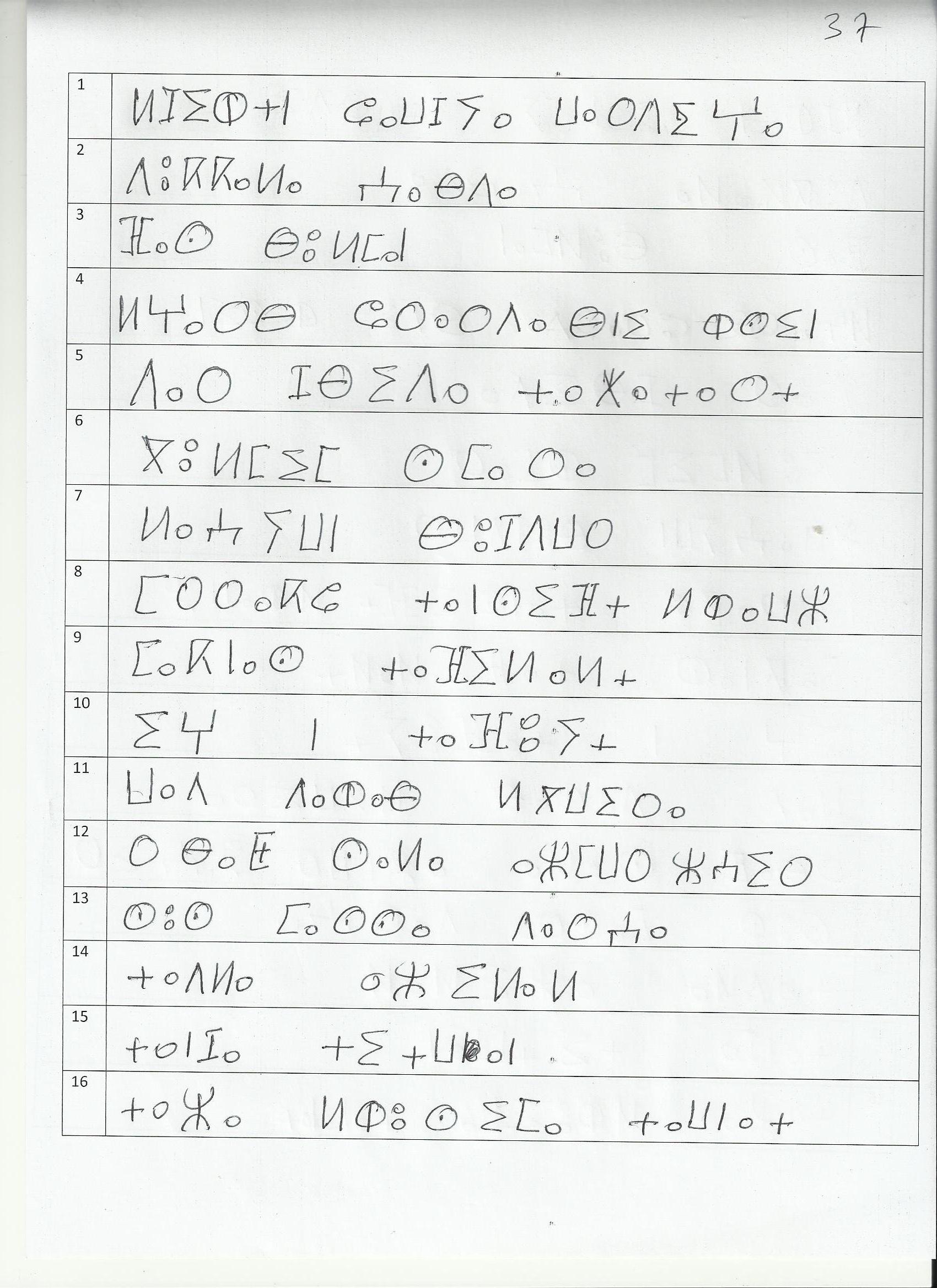

Supplement: Supplementary file 1 — Supplementary data [file mmc1.zip › EXAMPLE OF DATABASE/26.jpg]

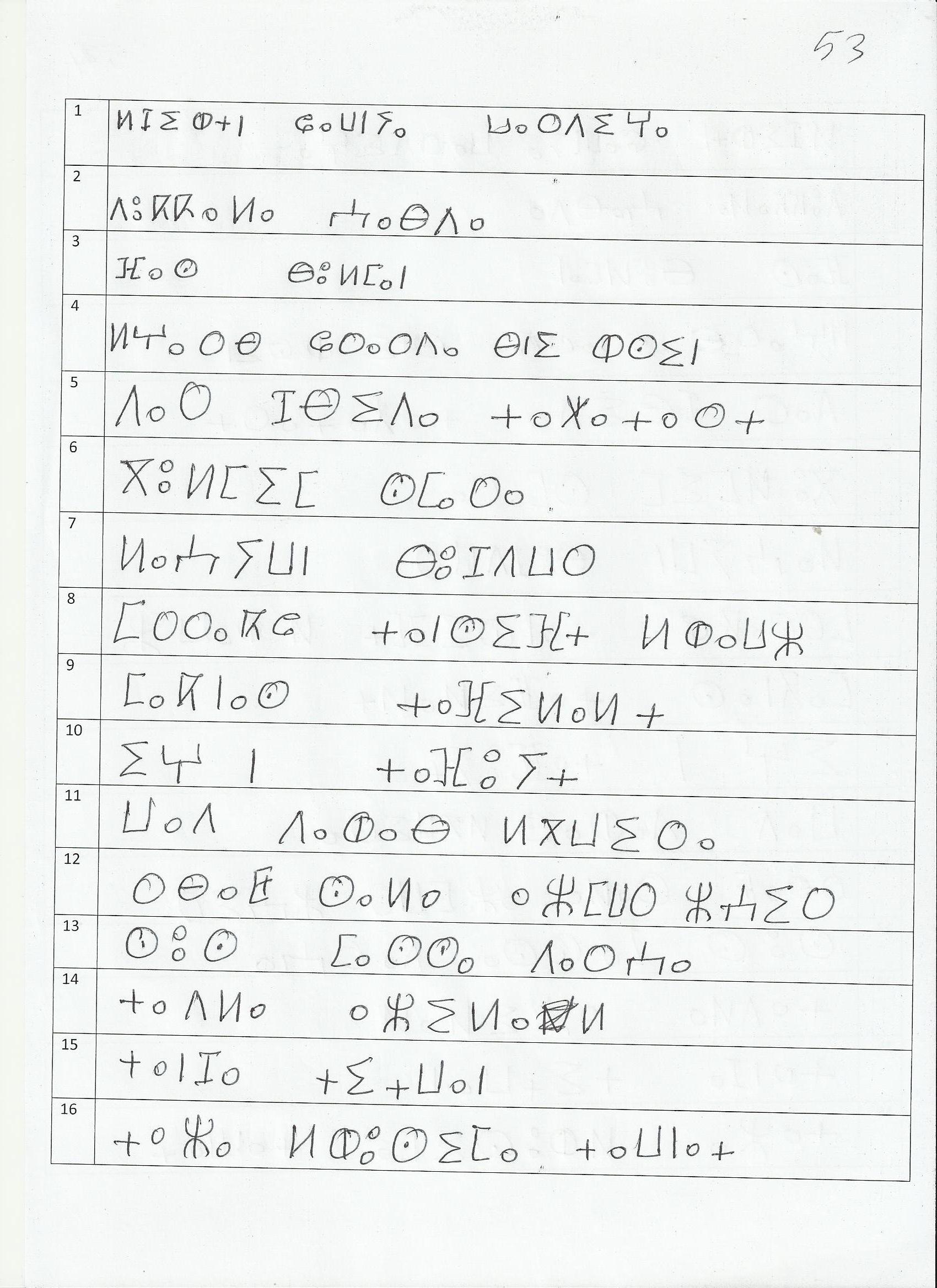

Supplement: Supplementary file 1 — Supplementary data [file mmc1.zip › EXAMPLE OF DATABASE/27.jpg]

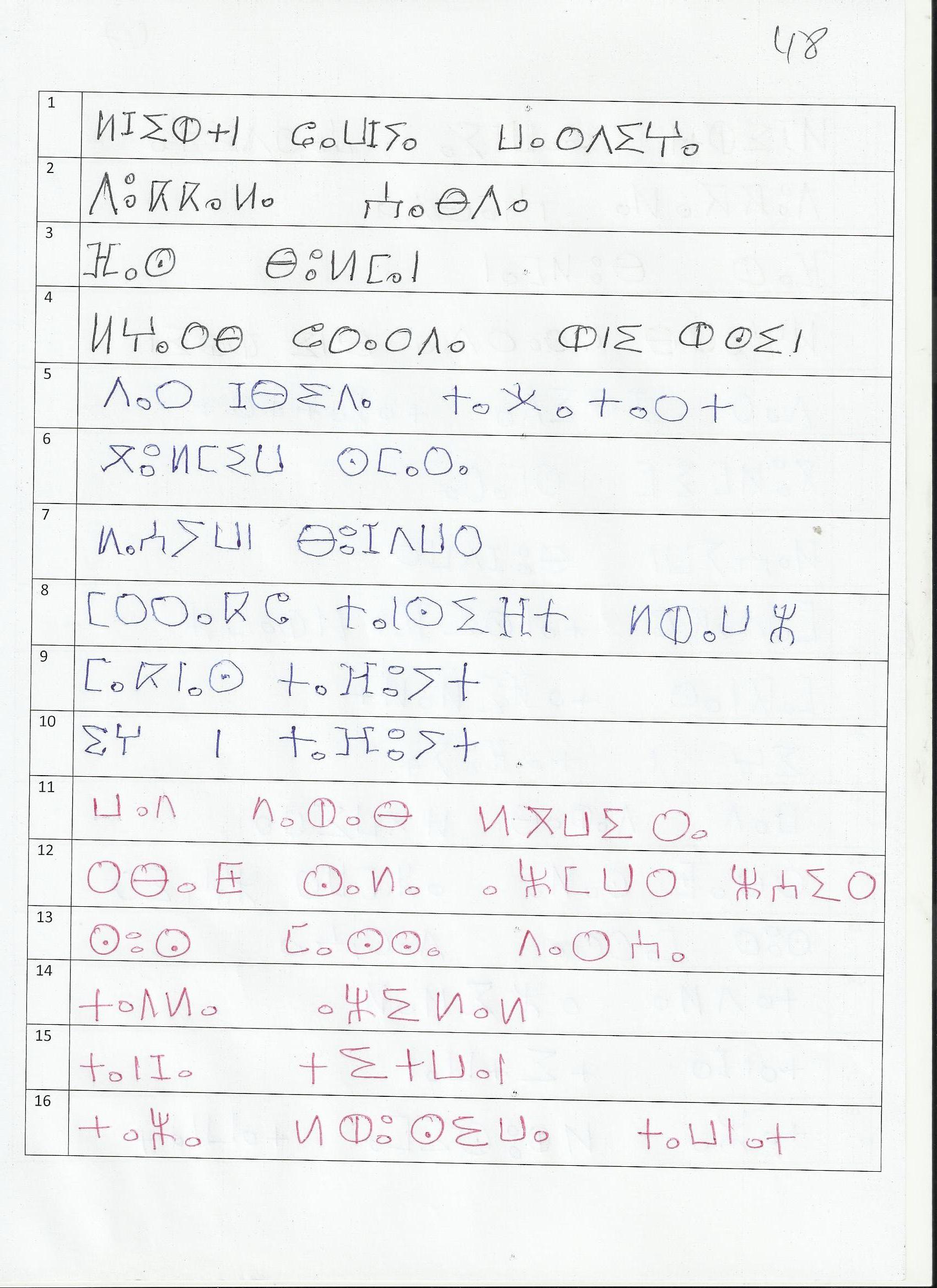

Supplement: Supplementary file 1 — Supplementary data [file mmc1.zip › EXAMPLE OF DATABASE/28.jpg]

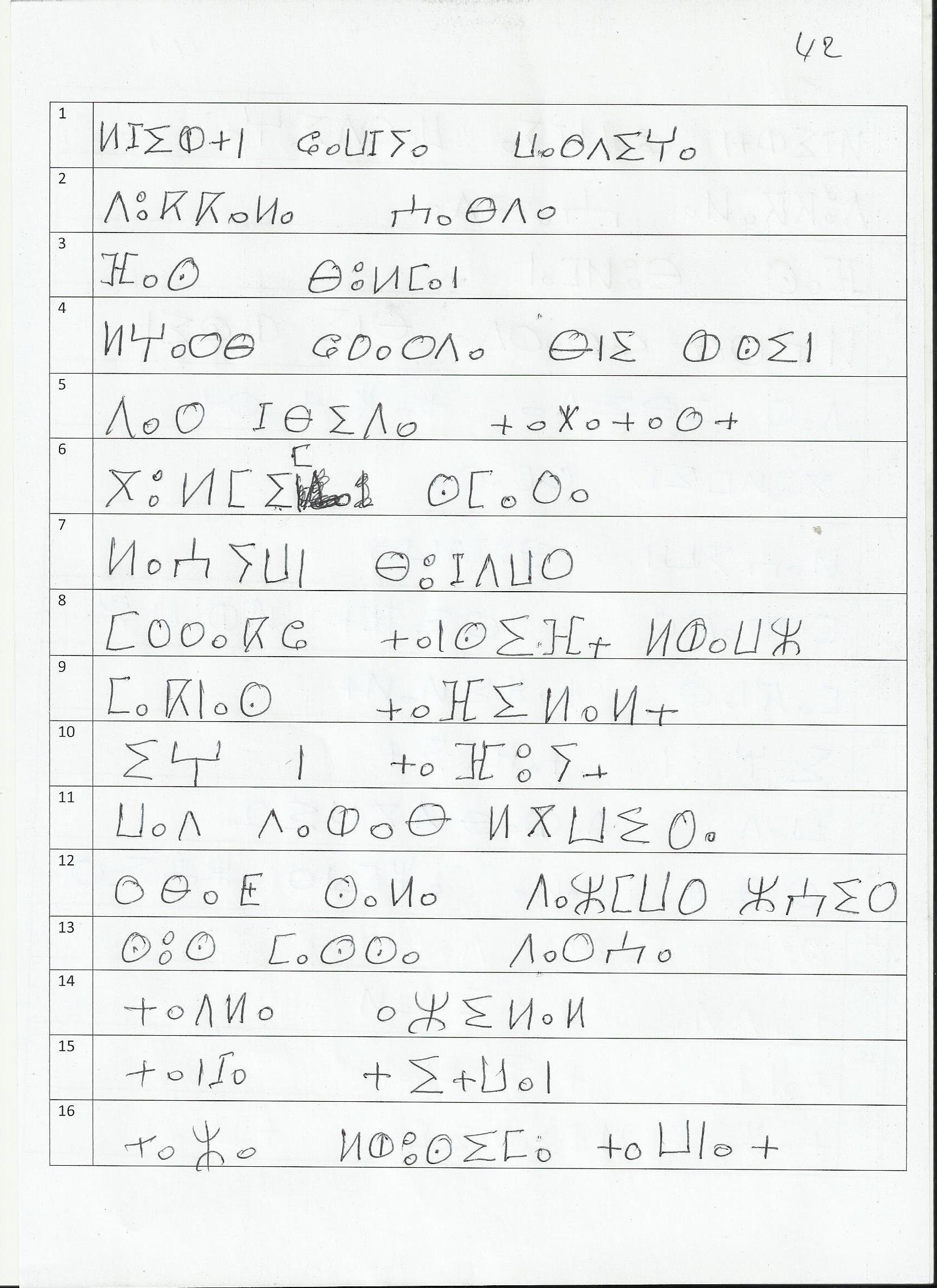

Supplement: Supplementary file 1 — Supplementary data [file mmc1.zip › EXAMPLE OF DATABASE/29.jpg]

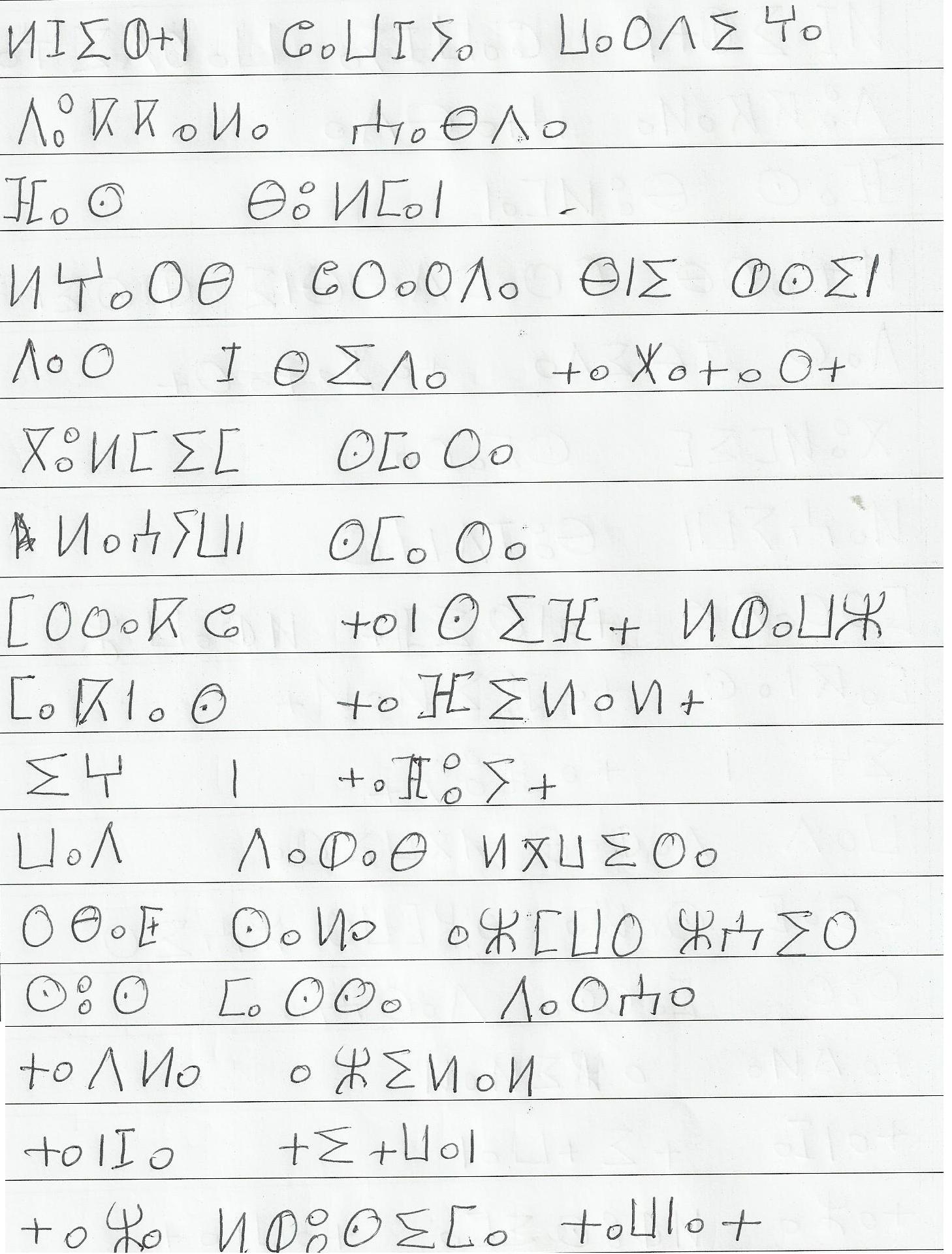

Supplement: Supplementary file 1 — Supplementary data [file mmc1.zip › EXAMPLE OF DATABASE/3.jpg]

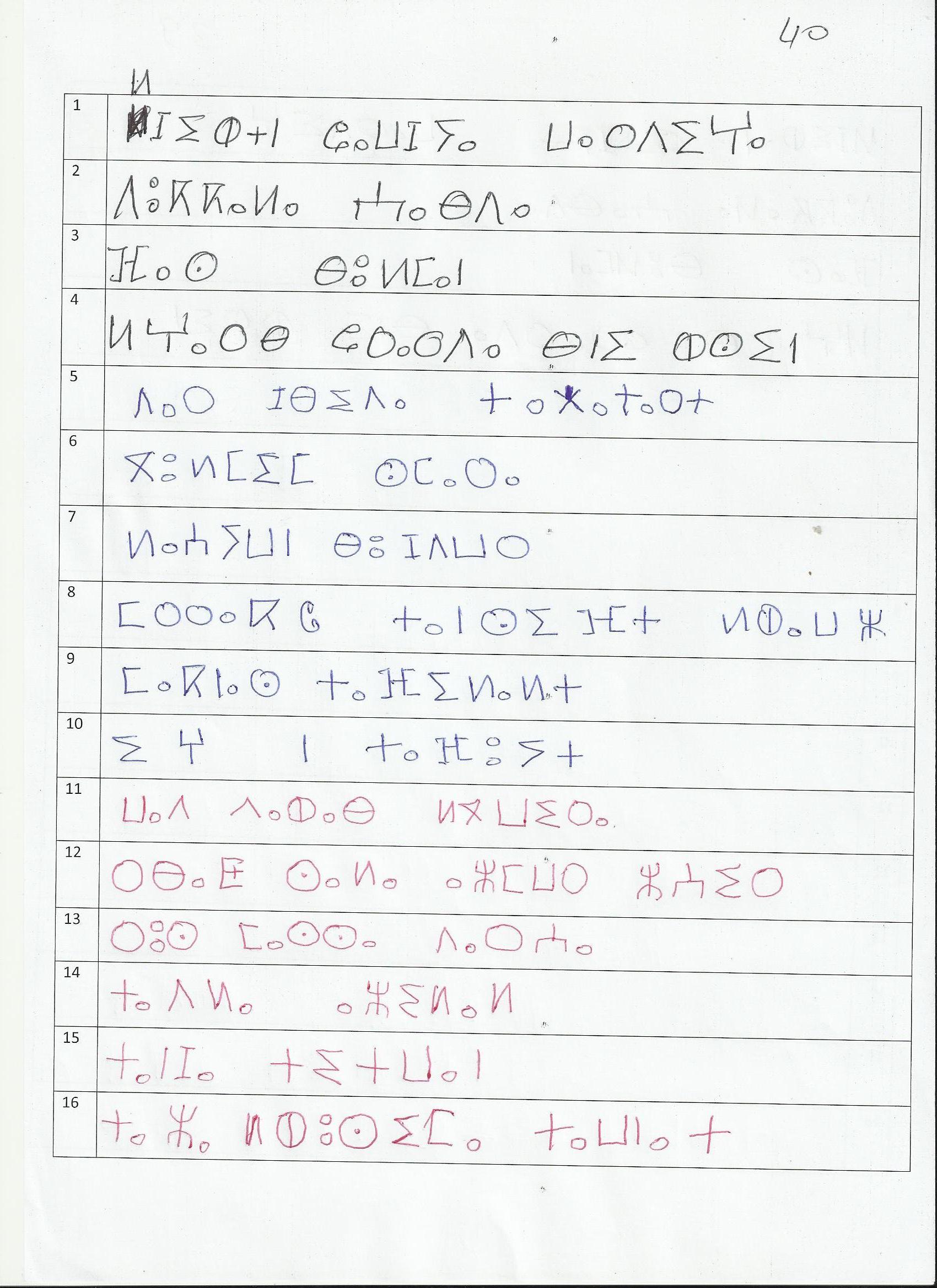

Supplement: Supplementary file 1 — Supplementary data [file mmc1.zip › EXAMPLE OF DATABASE/30.jpg]

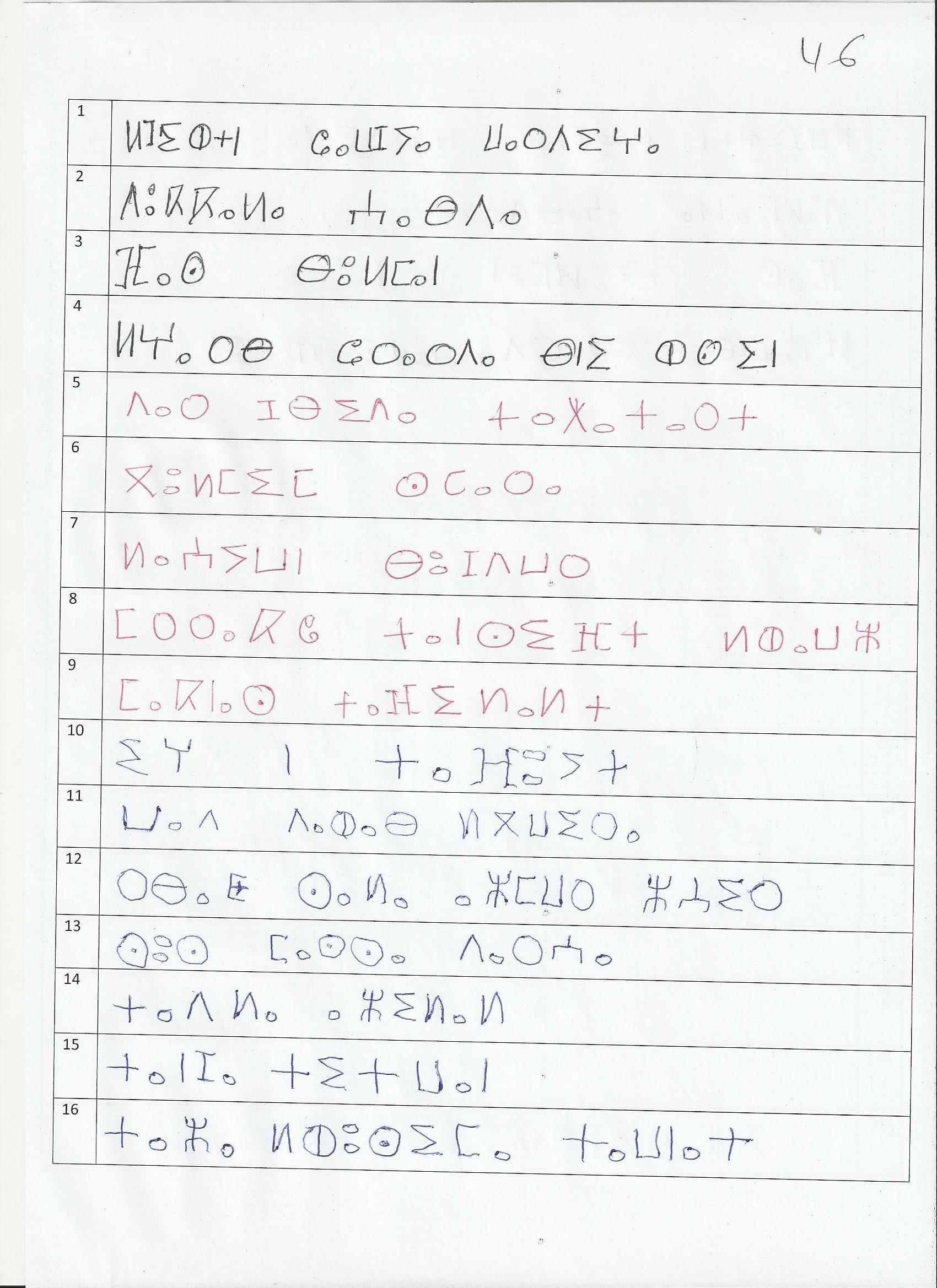

Supplement: Supplementary file 1 — Supplementary data [file mmc1.zip › EXAMPLE OF DATABASE/31.jpg]

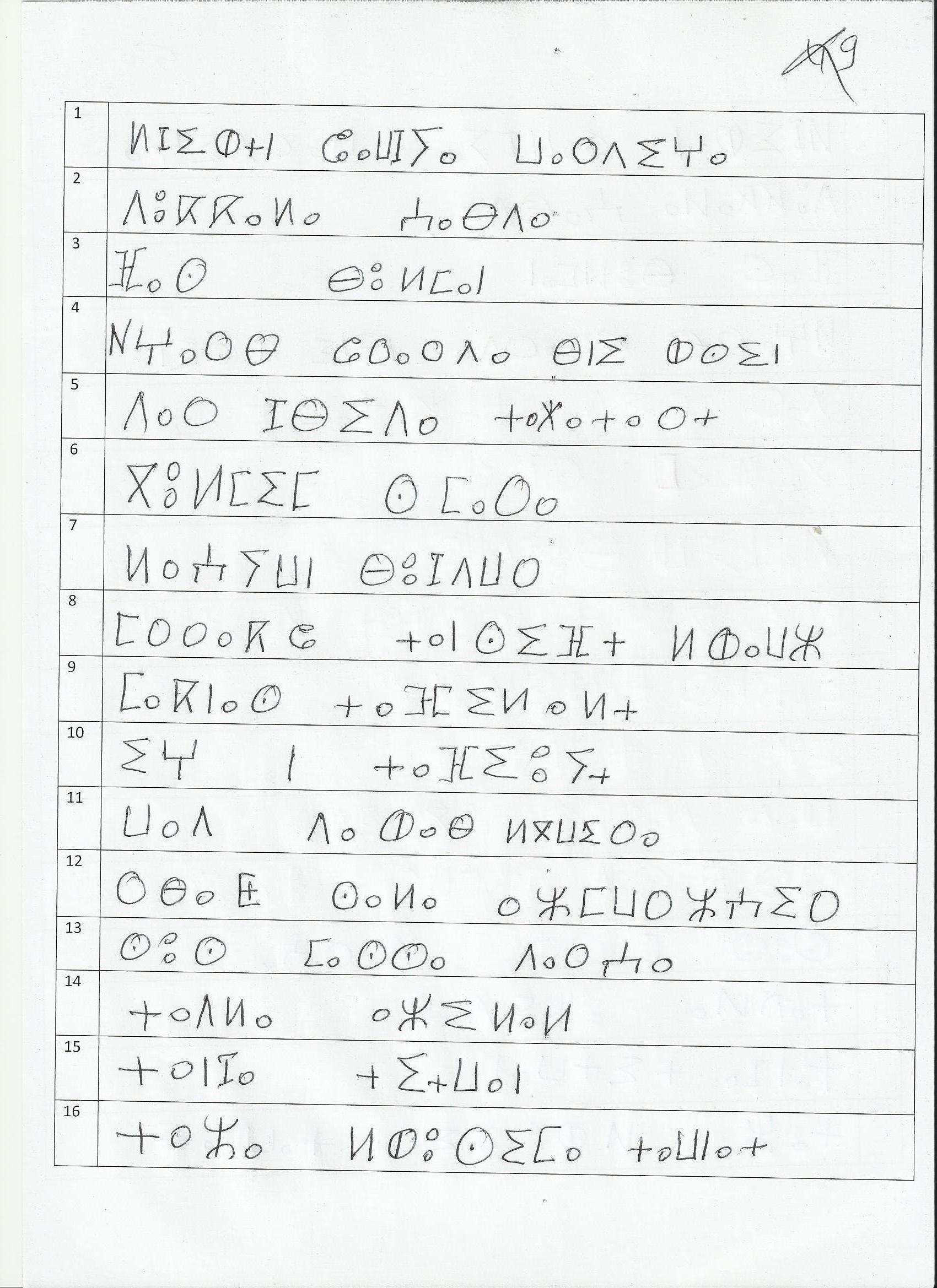

Supplement: Supplementary file 1 — Supplementary data [file mmc1.zip › EXAMPLE OF DATABASE/32.jpg]

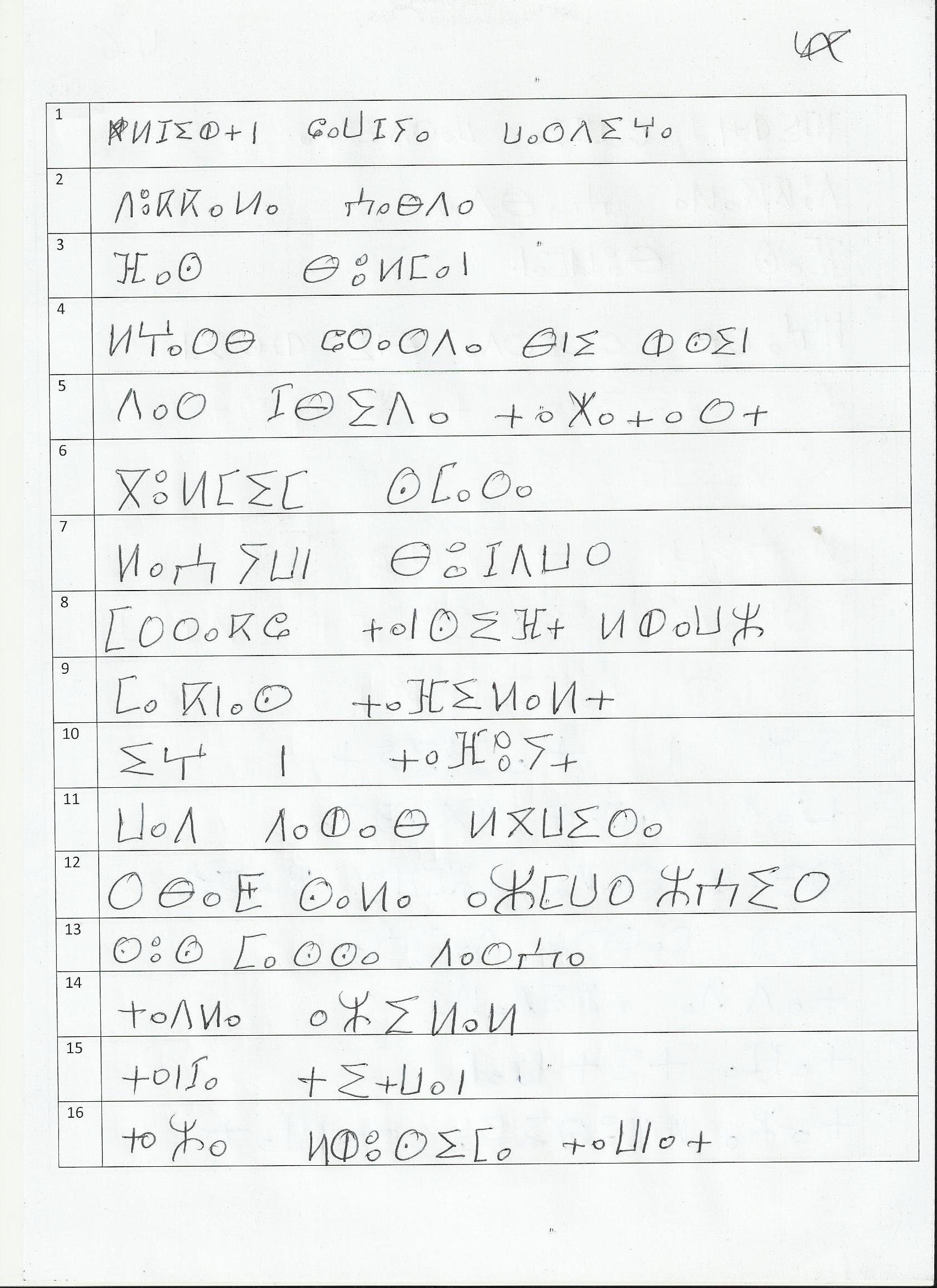

Supplement: Supplementary file 1 — Supplementary data [file mmc1.zip › EXAMPLE OF DATABASE/33.jpg]

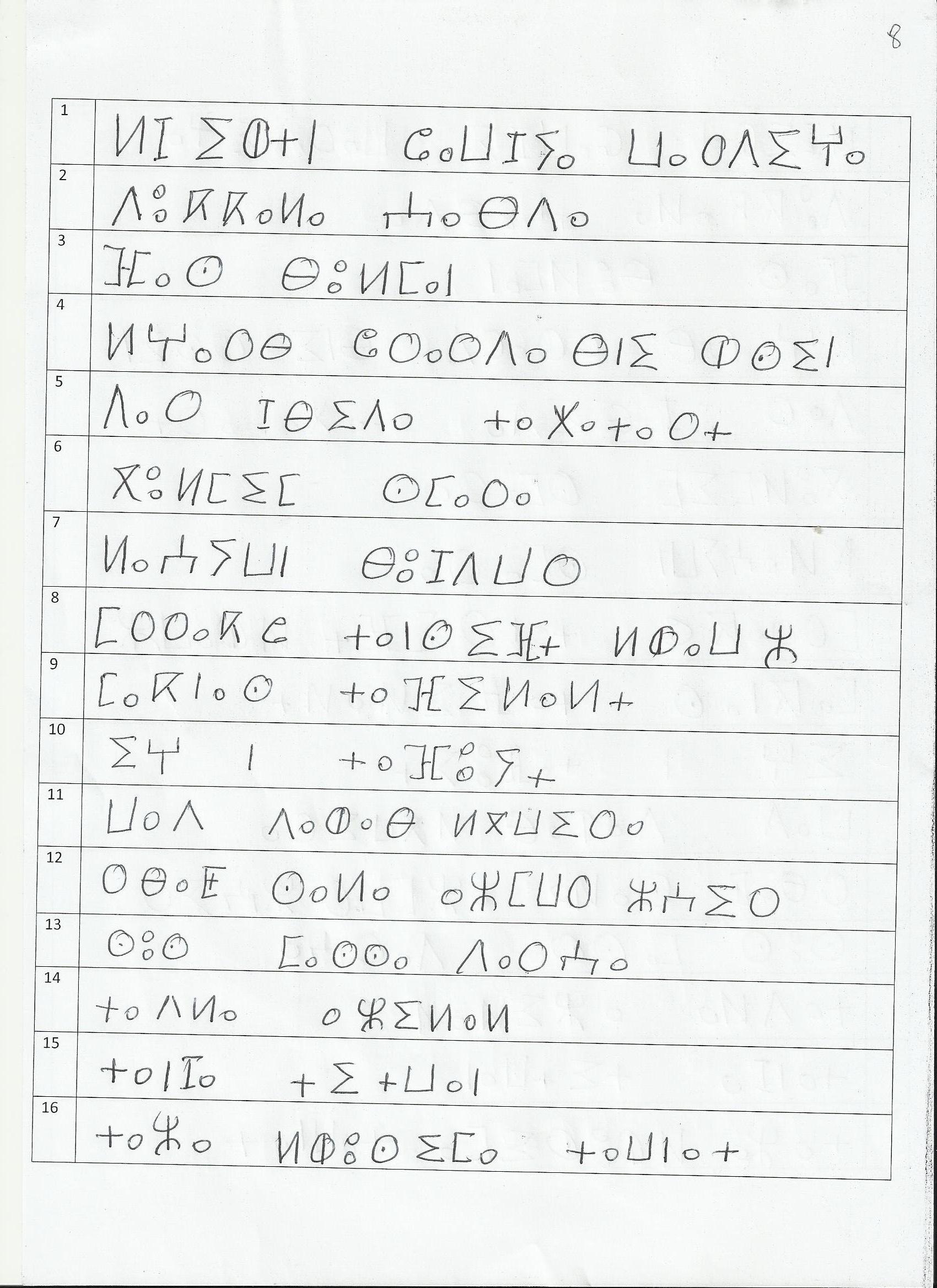

Supplement: Supplementary file 1 — Supplementary data [file mmc1.zip › EXAMPLE OF DATABASE/34.jpg]

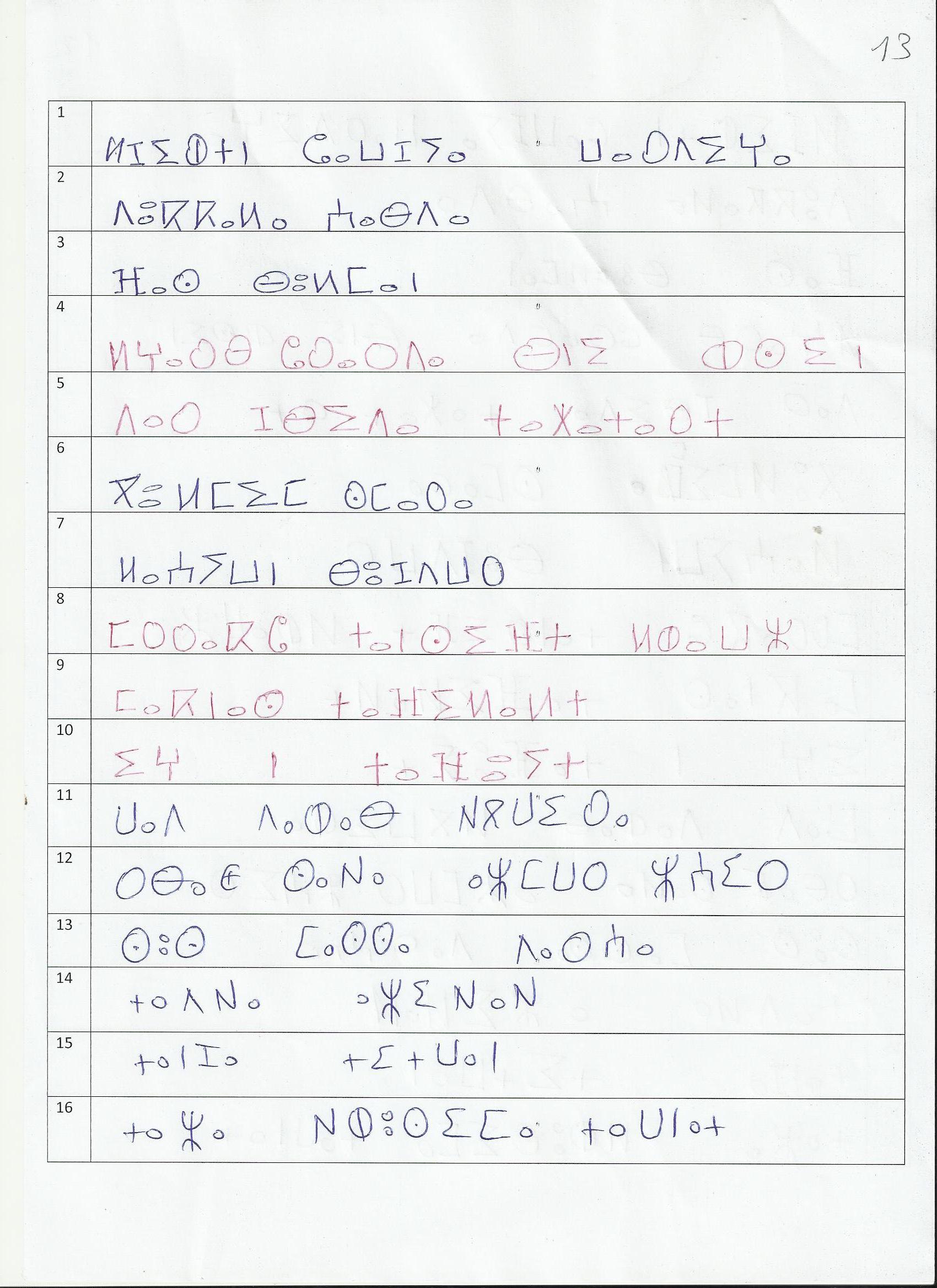

Supplement: Supplementary file 1 — Supplementary data [file mmc1.zip › EXAMPLE OF DATABASE/35.jpg]

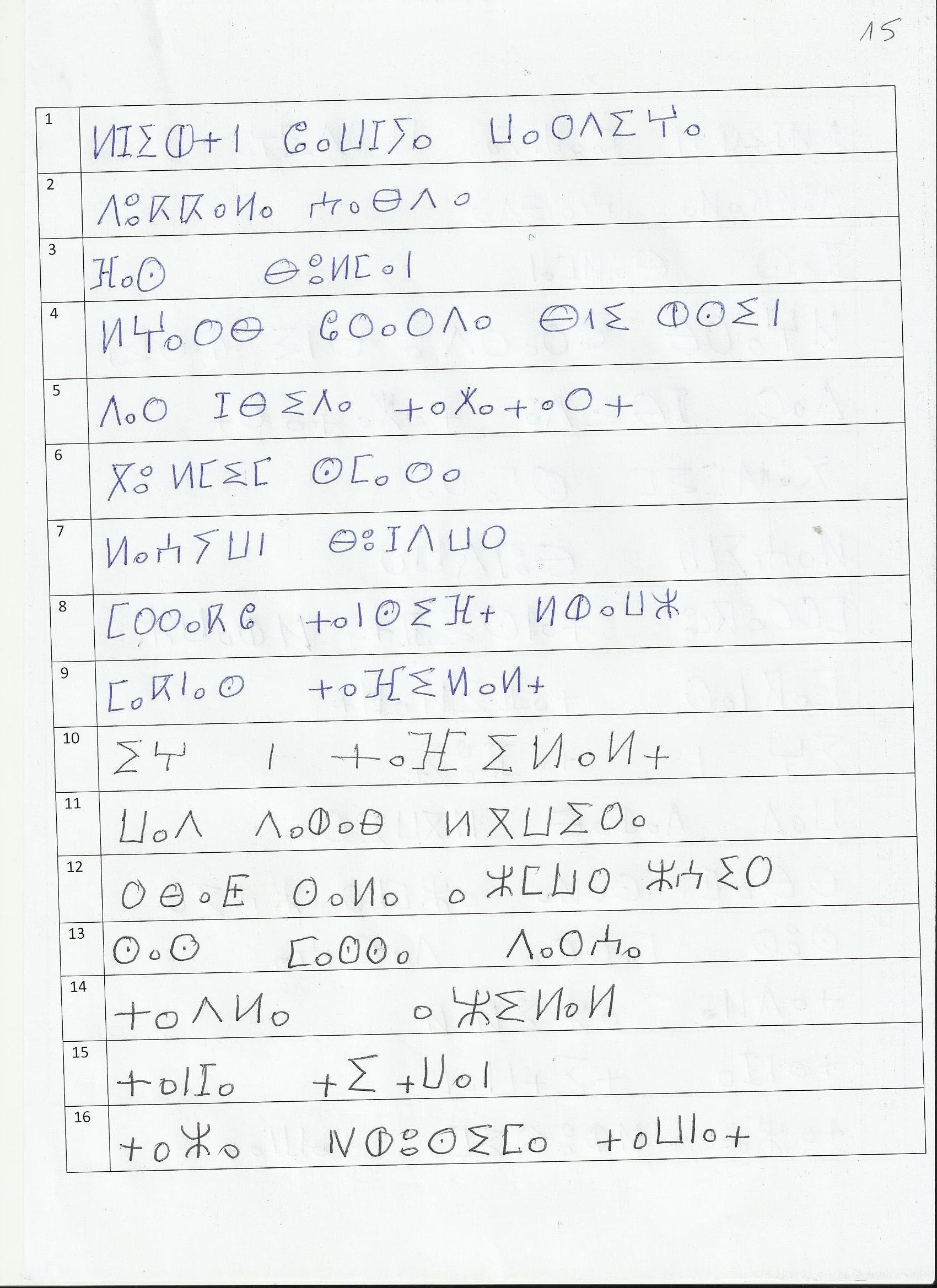

Supplement: Supplementary file 1 — Supplementary data [file mmc1.zip › EXAMPLE OF DATABASE/36.jpg]

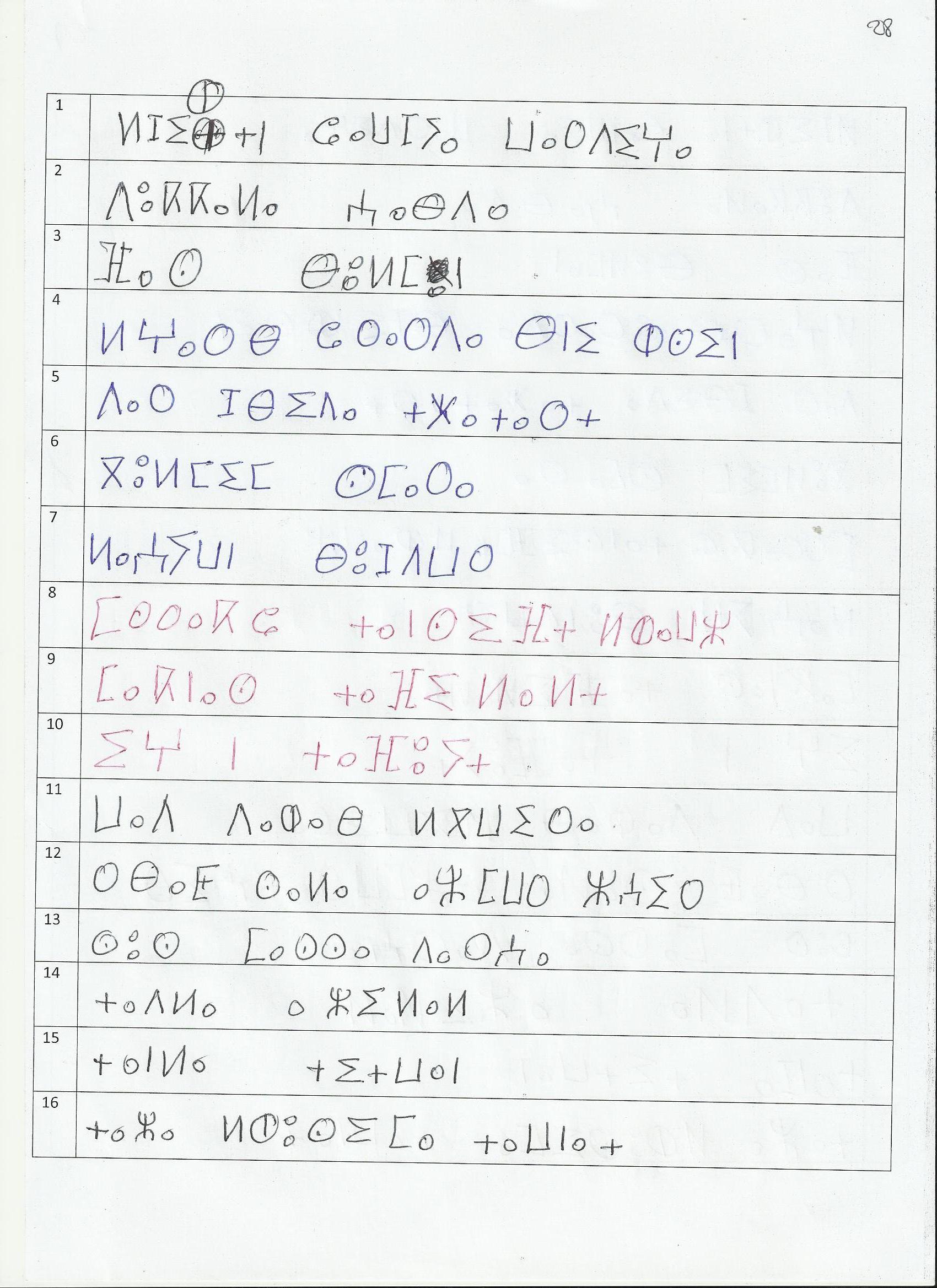

Supplement: Supplementary file 1 — Supplementary data [file mmc1.zip › EXAMPLE OF DATABASE/37.jpg]

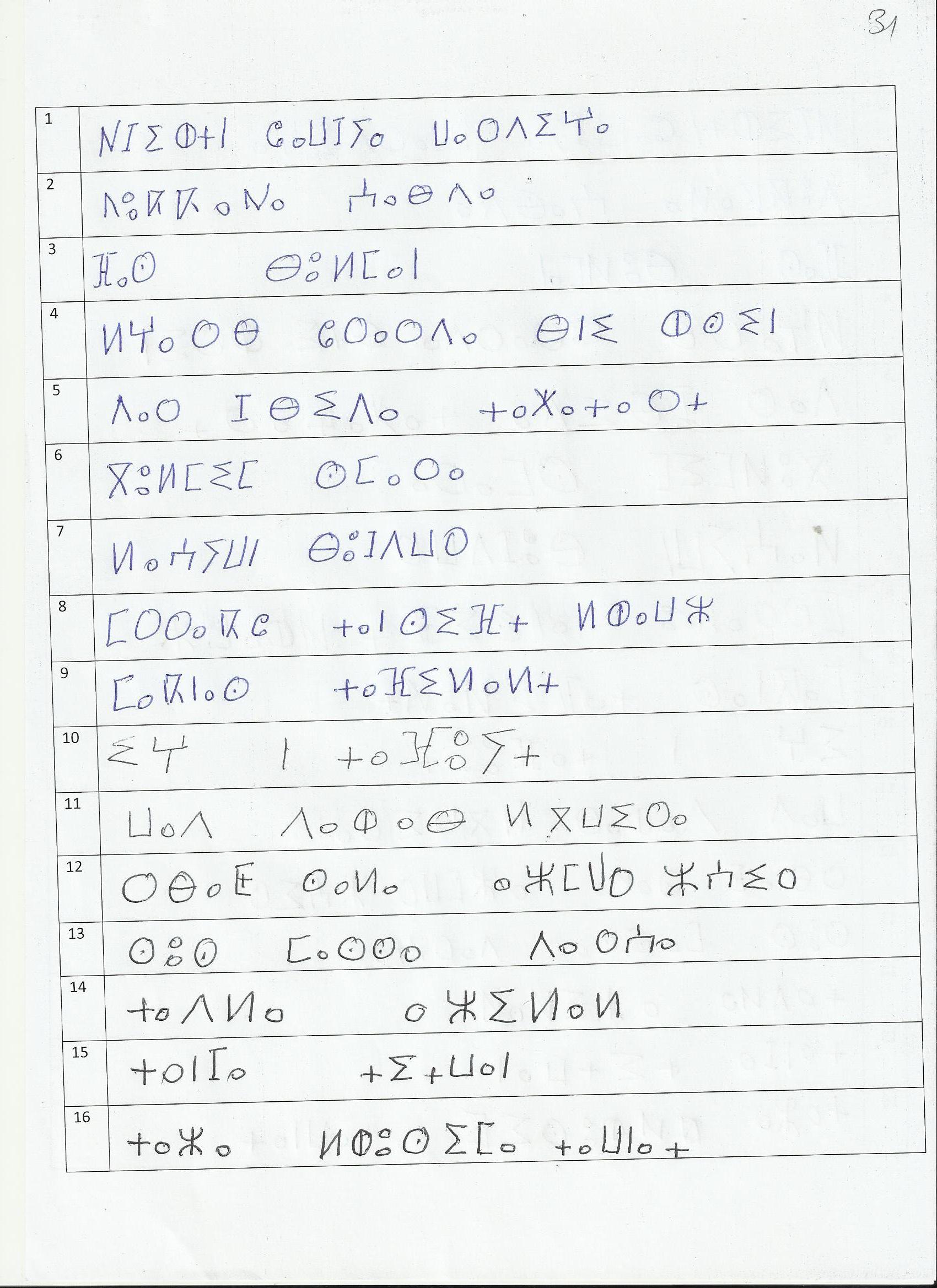

Supplement: Supplementary file 1 — Supplementary data [file mmc1.zip › EXAMPLE OF DATABASE/38.jpg]

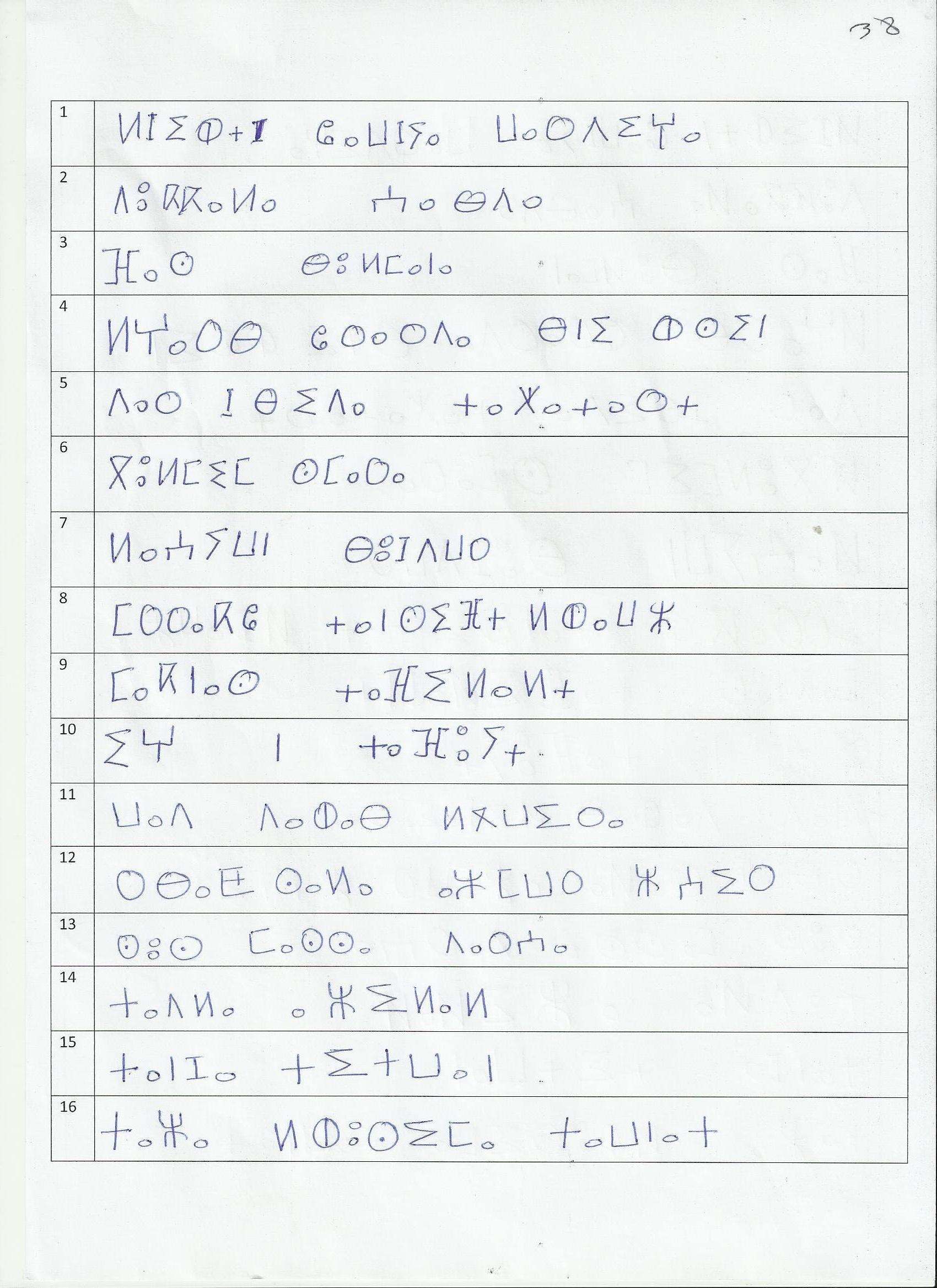

Supplement: Supplementary file 1 — Supplementary data [file mmc1.zip › EXAMPLE OF DATABASE/39.jpg]

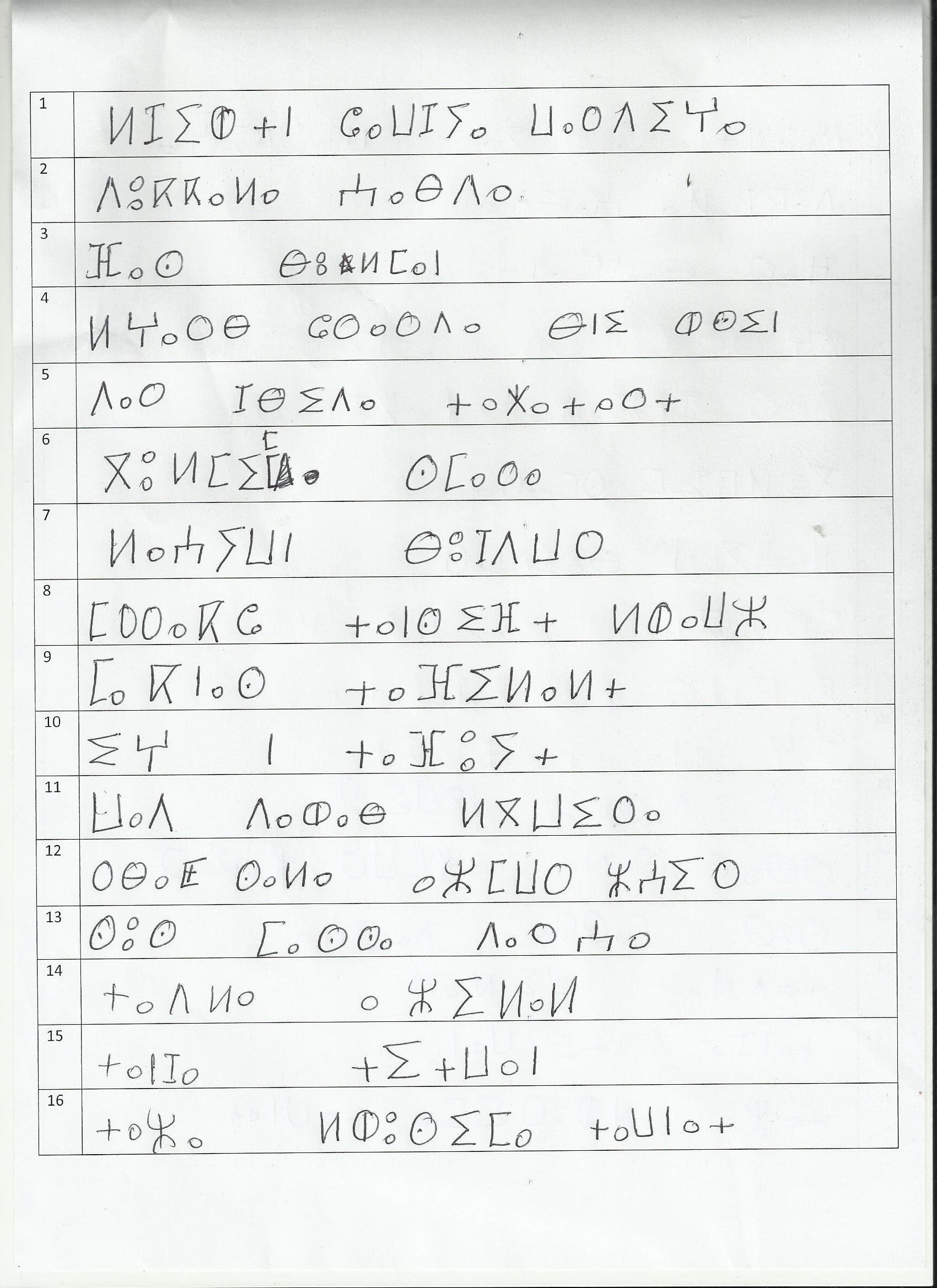

Supplement: Supplementary file 1 — Supplementary data [file mmc1.zip › EXAMPLE OF DATABASE/4.jpg]

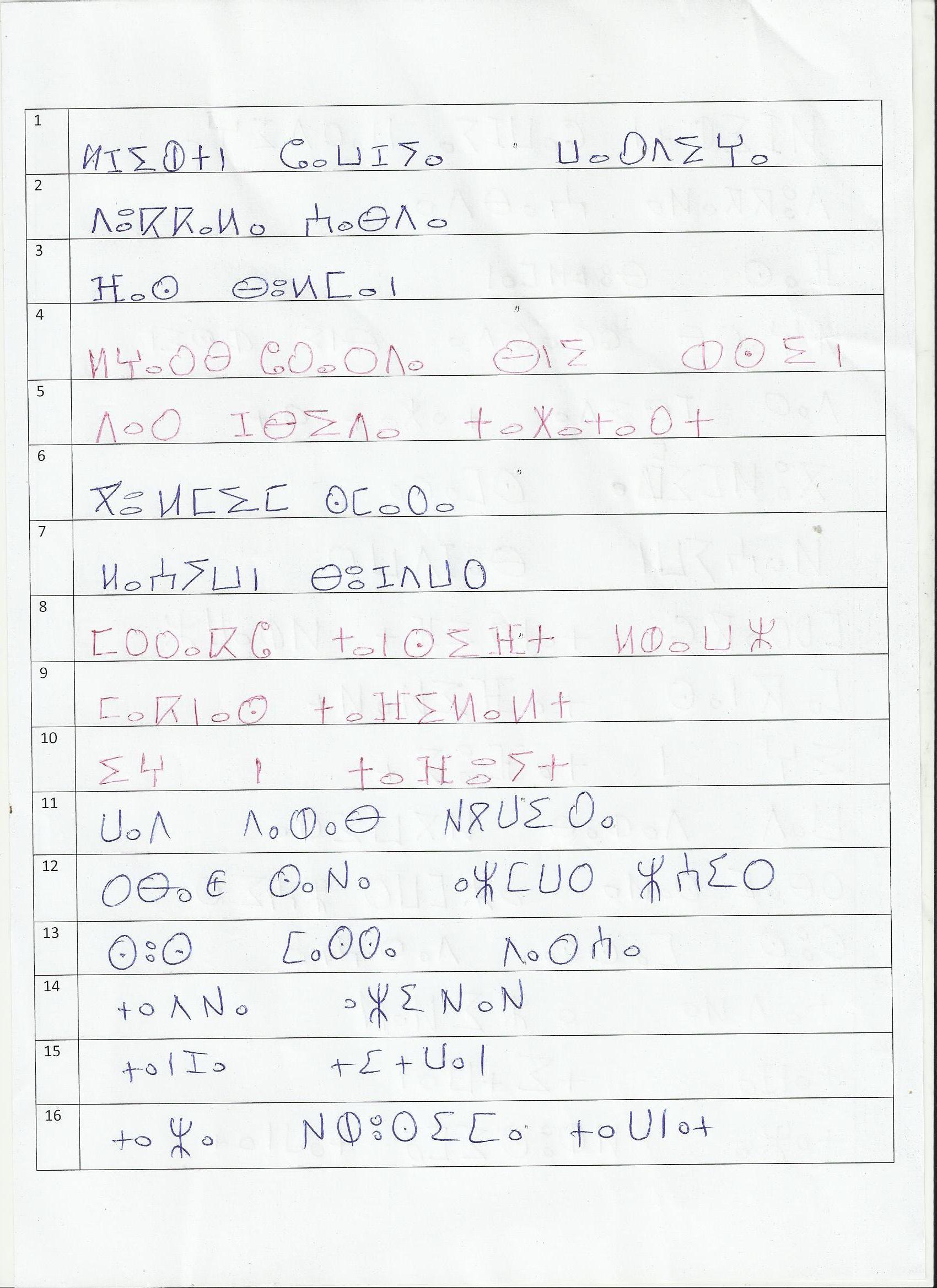

Supplement: Supplementary file 1 — Supplementary data [file mmc1.zip › EXAMPLE OF DATABASE/5.jpg]

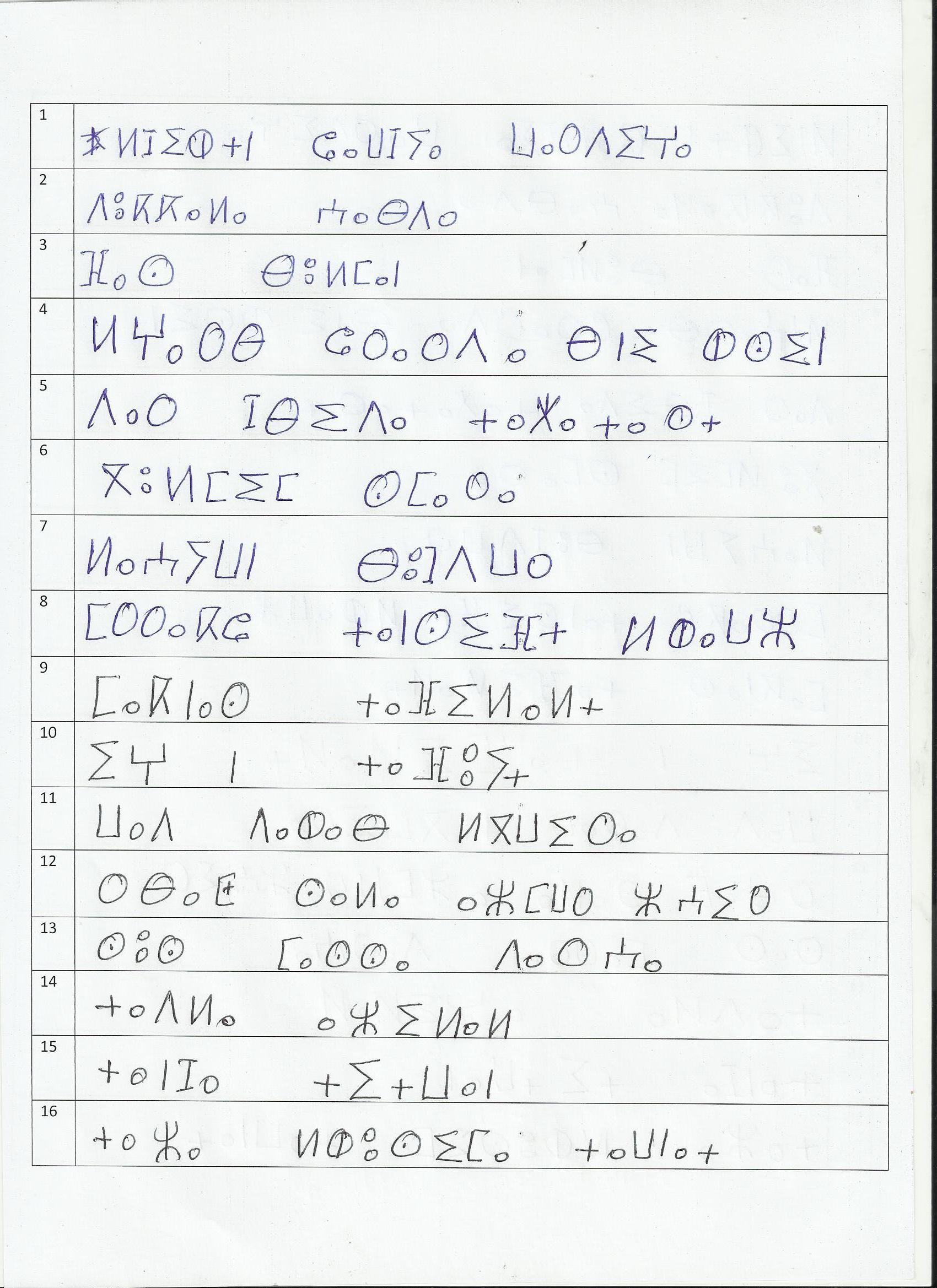

Supplement: Supplementary file 1 — Supplementary data [file mmc1.zip › EXAMPLE OF DATABASE/6.jpg]

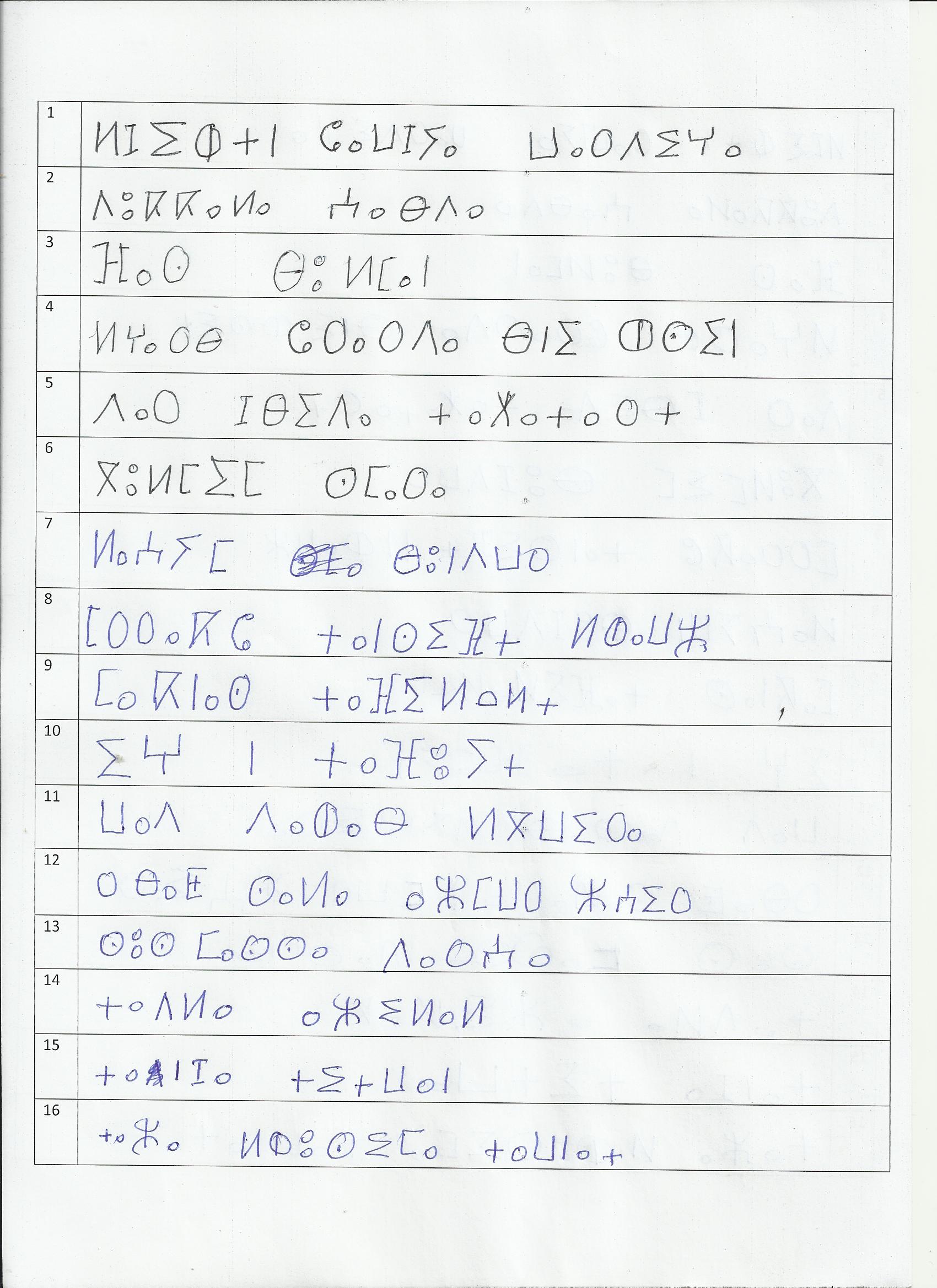

Supplement: Supplementary file 1 — Supplementary data [file mmc1.zip › EXAMPLE OF DATABASE/7.jpg]

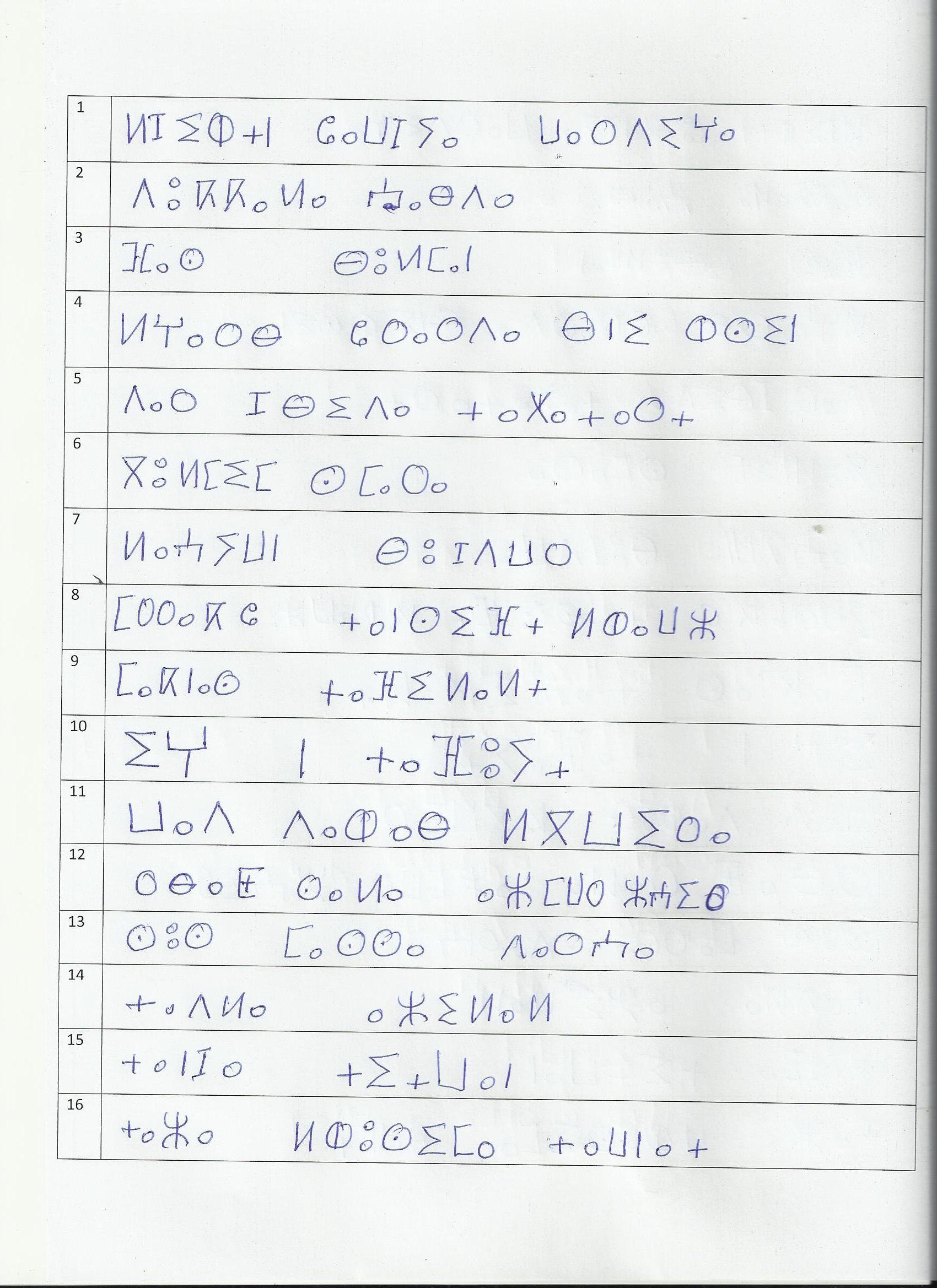

Supplement: Supplementary file 1 — Supplementary data [file mmc1.zip › EXAMPLE OF DATABASE/8.jpg]

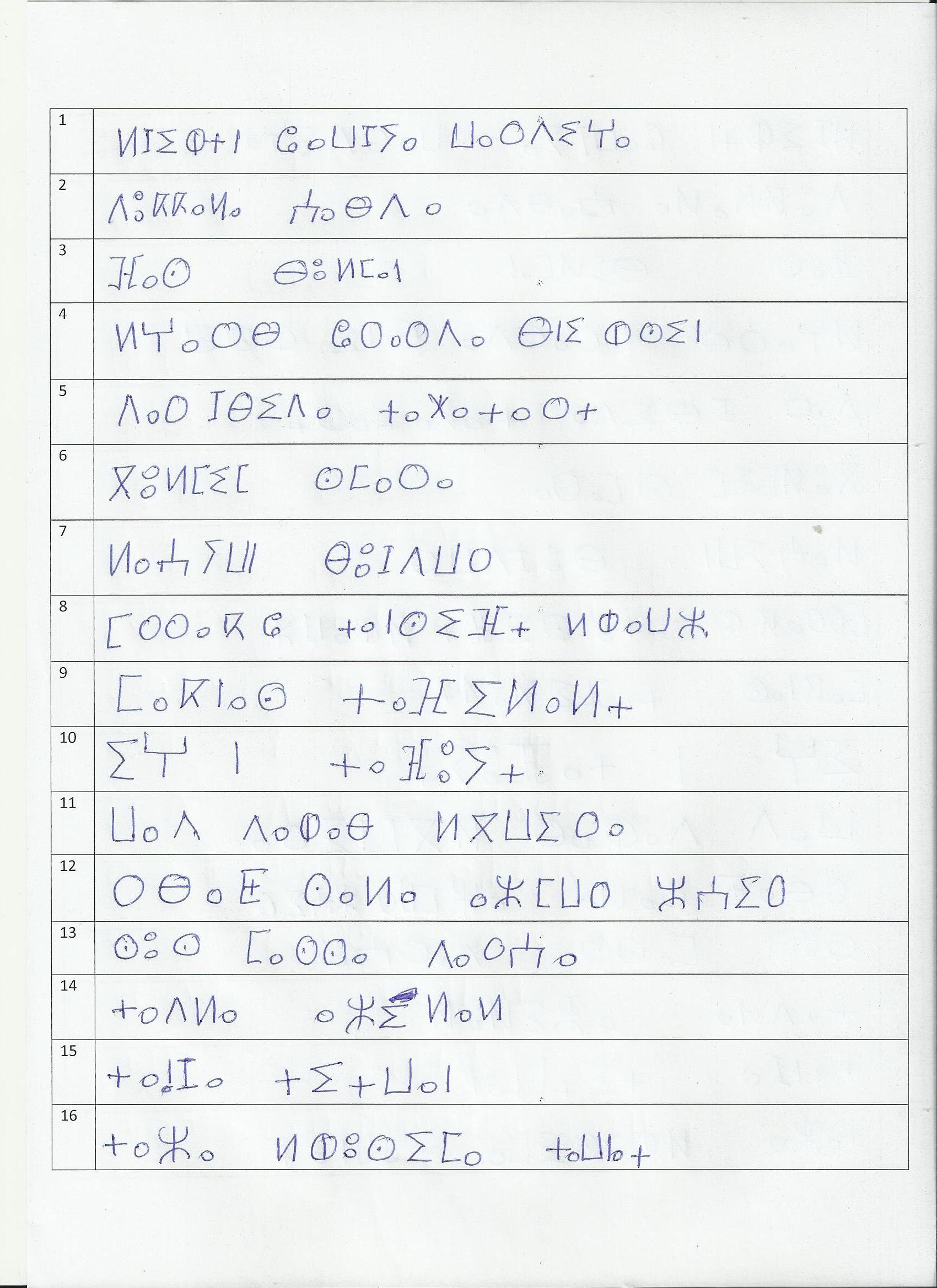

Supplement: Supplementary file 1 — Supplementary data [file mmc1.zip › EXAMPLE OF DATABASE/9.jpg]
