# Supplementary material for: Handwriting Moroccan regions recognition using Tifinagh character
Source: Data Brief. 2015 Jul 26;4:534–43. doi: 10.1016/j.dib.2015.07.018 (PMC4783523; doi:10.1016/j.dib.2015.07.018)
Supplement: Supplementary file 1 — Supplementary data [file mmc1.zip › Figure.doc]

***Fig 1****. Tifinaghs* *characters*

| *Printed Tifingh characters* | *Printed Latin characters* | *Printed Arabic characters* |
| --- | --- | --- |
|  | **A** | **أ** |
|  | **B** | **ب** |
|  | **C** | **ت** |
|  | **D** | **ث** |
|  | **E** | **ج** |
|  | **F** | **ح** |
|  | **G** | **خ** |
|  | **H** | **د** |
|  | **I** | **ذ** |
|  | **J** | **ع** |
|  | **K** | **غ** |
|  | **L** | **ف** |
|  | **M** | **ق** |
|  | **N** | **س** |
|  | **O** | **ش** |
|  | **P** | **ص** |
|  | **Q** | **ض** |
|  | **R** | ***ظ*** |
|  | **S** | **ط** |
|  | **T** | **ك** |
|  | **U** | **م** |
|  | **V** | **ن** |
|  | **W** | **ل** |
|  | **X** | **و** |
|  | **Y** | **ي** |
|  | **Z** | **ر** |
|  | **--** | **ز** |
|  | **--** | **ه** |
|  | **--** | **--** |
|  | **--** | **--** |
|  | **--** | **--** |
|  | **--** | **--** |
|  | **--** | **--** |

***Fig 2****. Comparison between the Tifinagh, Arabic and Latin characters*

***Fig 3****. Sixteen Tifinagh region*

***Fig 4****. Comparison between Sixteen Moroccan region with Tifinagh, Arabic and French langugae*

**Median filter**

**Normalization**

**Thresholding**

**Thinning**

**Pretreatment**

**Centering**

**Skeletonization**

**Segmentation**

***Fig 5****. The pretreatment steps*

***Fig. 6****. The original image*

***Fig. 7****. Graphical representation of segmentation column*

***Fig.8****. The segmentation in columns.*

***Fig. 9****. The square zoning method*

*Vector Of Extraction = [Number Of White Pixels In Zone 1, ….*

*…... Number Of White Pixels In Zone9]*

| *Zone 1* | *Zone 2* | *Zone 3* |
| --- | --- | --- |
| *Zone 4* | *Zone 5* | *Zone 6* |
| *Zone 7* | *Zone 8* | *Zone 9* |

***Fig. 10****. Processes of feature extraction by square zoning*

***Fig. 11****. The triangular zoning method*

*Zone 2*

*Zone 1*

*Zone 3*

*Zone 4*

*Zone 5*

*Zone 6*

*Zone 7*

*Zone 8*

*Vector Of Extraction = [Number Of White Pixels In Zone1, ….*

*…... Number Of White Pixels In Zone9]*

***Fig. 12****. Processes of feature extraction by triangular zoning*

***Fig. 13****. The determination of optimal hyperplane, vectors supports, maximum Marge and valid hyperplanes.*

***Fig. 14****. The multi-layer perceptron*

***Fig. 15****. The graphical representation of recognition rate τr of each region with all methods*

***Fig. 16****. The graphical representation of recognition rate τ_r_ of all feature extraction methods*

***Fig. 17****. The graphical representation of recognition rate τ_r_ of square zoning method and SVM classifier*
