# Supplementary material for: Handwriting Moroccan regions recognition using Tifinagh character
Source: Data Brief. 2015 Jul 26;4:534–43. doi: 10.1016/j.dib.2015.07.018 (PMC4783523; doi:10.1016/j.dib.2015.07.018)
Supplement: Supplementary file 1 — Supplementary data [file mmc1.zip › Table.doc]

All values of the recognition rate for each region **τ_r_** (given in %) and also those of the global rate recognition **τ_g_** of all 16 regions (given in %) which we have obtained in the following table:

**Table 1.** The obtained recognition rates **τ_r_** and **τ_g_** by each hybrid method and each classifier.

| **Regions** | **Neural networks** | | **Support vectors machines** | |
| --- | --- | --- | --- | --- |
|  | **Square zoning** | **triangular zoning** | **Square zoning** | **triangular zoning** |
|  | 70,00 | 67,57 | 82,00 | 74,49 |
|  | 79,13 | 73,4 | 80,00 | 75,18 |
|  | 60,00 | 60,74 | 83,00 | 80,34 |
|  | 55,09 | 53,48 | 76,67 | 70,61 |
|  | 65,21 | 64,00 | 74,00 | 70,78 |
|  | 63,25 | 65,85 | 69,00 | 66,60 |
|  | 50,18 | 50,97 | 68,67 | 65,00 |
|  | 69,66 | 65,93 | 71,67 | 70,56 |
|  | 64,46 | 61,71 | 67,00 | 63,60 |
|  | 71,31 | 67,40 | 72,00 | 70,96 |
|  | 73,00 | 71,43 | 74,00 | 71,73 |
|  | 66,11 | 64,84 | 67,57 | 69,41 |
|  | 68,67 | 62,69 | 70,40 | 69,00 |
|  | 73,37 | 69,29 | 72,74 | 70,44 |
|  | 67,45 | 66,04 | 69,48 | 61,33 |
|  | 69,26 | 68,34 | 81,4 | 74,18 |
| **τ_g_** | **66,64** | **64,61** | **73,73** | **70,26** |

***Fig. 1****. The graphical representation of recognition rate τ_r_ of each region with all methods*

***Fig. 2****. The graphical representation of global recognition rate τ_g_ of all feature extraction methods*

***Fig. 3****. The graphical representation of recognition rate τ_r_ of square zoning method and SVM classifier*
